# Supplementary material for: Low atmospheric CO2 levels before the rise of forested ecosystems
Source: Nat Commun. 2022 Dec 20;13:7616. doi: 10.1038/s41467-022-35085-9 (PMC9768202; doi:10.1038/s41467-022-35085-9)
Supplement: Supplementary file 1 — Supplementary information [file 41467_2022_35085_MOESM1_ESM.pdf]

Supplementary Materials for

Low atmospheric CO_2 levels before the rise of forested ecosystems

Tais W. Dahl[1,2*], Magnus A. R. Harding[1,3], Julia Brugger[4,5], Georg Feulner[4], Kion Norrman[6], Barry H. Lomax[7], and Christopher K. Junium[8].

[1]Globe institute, University of Copenhagen; Øster Voldgade 5-7, Copenhagen, Denmark.

[2]State Key Laboratory of Geological Processes and Mineral Resources, China University of Geosciences; Wuhan, China.

[3]Sino-Danish College (SDC), University of Chinese Academy of Sciences, Beijing, China.

[4]Earth System Analysis, Potsdam Institute for Climate Impact Research, Member of the Leibniz Association; Potsdam, Germany.

[5]Senckenberg Biodiversity and Climate Research Centre; Frankfurt am Main, Germany.

[6]Center for Integrative Petroleum Research, King Fahd University of Petroleum and Minerals; Dhahran, Saudi Arabia.

[7]School of Biosciences, University of Nottingham, Sutton Bonington Campus; Leicestershire, UK.

[8]Department of Earth and Environmental Sciences, Syracuse University; Syracuse, New York, USA.

Correspondence to: tais.dahl@sund.ku.dk

The file includes:
Supplementary Text
Supplementary figs. 1 to 21
Supplementary Tables 1 to 7

Table of Contents

Section S1. Geological setting and samples 3
S.1.1 Fossil locality and age 3
S1.2 Sample characterization 4
S1.3 Characterization of the fossilized plant tissue 8
S1.3.1 Carbon compounds 8
S1.3.2 Carbon isotopes 9
Section S2. Carbon isotope fractionation, stomata size and density in terrestrial plants versus ambient CO_2 level 11
S2.1 The gas-leaf exchange model – constraining CO2 assimilation rate and stomatal conductance in the Baragwanathia flora from observed C isotope fractionation, stomata density and stomatal pore size. 11
S2.2. Numerical solution to the mathematical problem 12
S2.3 Calibration in extant lycophytes 12
S2.3.1 Calibration to Huperzias 12
S2.4 Application to Devonian lycophytes 14
S2.5 Sensitivity analyses 19
S2.6. Reconstructing atmospheric pCO_2 from other Devonian lycophytes 22
S2.7 The effect of water utilization efficiency 25
S2.8 Carbon isotope composition of the Early Devonian atmosphere 27
Section S3. Error propagation for other atmospheric pCO_2 proxies 28
S3.1 Stomatal proxies for paleoatmospheric CO_2 28
S3.2 Pedogenic goethite as proxy for palaeoatmospheric pCO_2 29
S3.3 Paleosol carbonate proxy for paleoatmospheric CO_2 30
Section S4. Early Devonian Climate model. 32
S4.1 Early Devonian climate state 32
S4.2. Sea Surface Temperatures in the Early and Middle Devonian 35
S4.3 Mean Annual Precipitation at the Devonian fossil localities 37
Section S5. Revised COPSE model for the Mid-Paleozoic 38
Supplementary references 43

Supplementary text

Section S1. Geological setting and samples

The Early Devonian palaeo-CO_2 constraints from 66 plant fossils from 10 sites spanning 28.6 million years of geological time with representative isotope data of a larger number of plant fragments. We report new isotope data from the *Baragwanathia* flora in Australia and characterize the geological setting and samples below.

S.1.1 Fossil locality and age

Macrofossils of the early land flora from the Yea district in central Victoria, Australia, occur both in Late Silurian sediments (Gorstian Ludlow; 427.4-425.6 Ma) and sediments of Early Devonian age (Early Pragian-Early Emsian; 410.8–407.6 Ma)[1–4]. Due to structural folding and scarcity of diagnostic age markers in the area, it has proven challenging to assign an age to fossils from localities where only one of the plant assemblages have been found. However, the samples used in this study have been independently dated and come from the younger assemblage, as detailed below.

Plant macrofossils from the fossil collection of Museums Victoria were investigated using carbon isotope analysis. The specimens were collected by Isabel Cookson at Mt Pleasant from a cutting in "the old road" 1.25 mi south of Alexandra towards Thornton (for maps, see Earp 2019). At this locality, she described several plant macrofossils including *Baragwanathia longifolia* (in #15154, #15173, #15174, #15183), *Hedeia corymbose (cf Yarravia)*, and *Zosterophyllum australianum*[2,5]. The samples contain numerous small plant fragments in which many fossils preserve thick brown to black mineralization containing organic tissue well suited for organic characterization and carbon isotope analysis (described in more detail in S1.3).

In 1935, Isabel Cookson acknowledged that the age of the plant assemblage at Mt Pleasant was an unresolved stratigraphic problem. Since then, graptolite biostratigraphy has settled that the fossil assemblage from Mt Pleasant is Early Devonian in age and roughly coeval with the younger plant assemblages observed near Yea (~30 km to the west) and Matlock (~75 km to the southeast).

The wider Alexandra area is a broad northwest-southeast striking synclinorium with a large number of impersistent folds 10s to 100s of meters apart and generally only traceable for kilometers. Closer to the sample location, the structure is that of an anticlinorium passing through the town of Alexandra. The Mt Pleasant road cutting sits on the uniformly dipping western limb of the anticlinorium[4]. Supplementary figure 1 shows the stratigraphy across the anticlinorial folding where Mt Pleasant road cuts through thin bedded siltstone. Cookson described the plant remains from fine-grained sandstone beds, which have been identified at the P4 interval (Supplementary fig. 1). At this level, the early land plant *Hedeia corymbose* is found together with early Devonian index fossils (*Uncinatograptus sp.* cf. *U. thomasi* and *Nowakia sp.* ex gr. *N. acuaria*). This confines the age of the Mt Pleasant land plant fossils to the Pragian or earliest Emsian, and we can assign an age of the studied samples of ~409.1 ±1.5 Ma according to GTS2020.

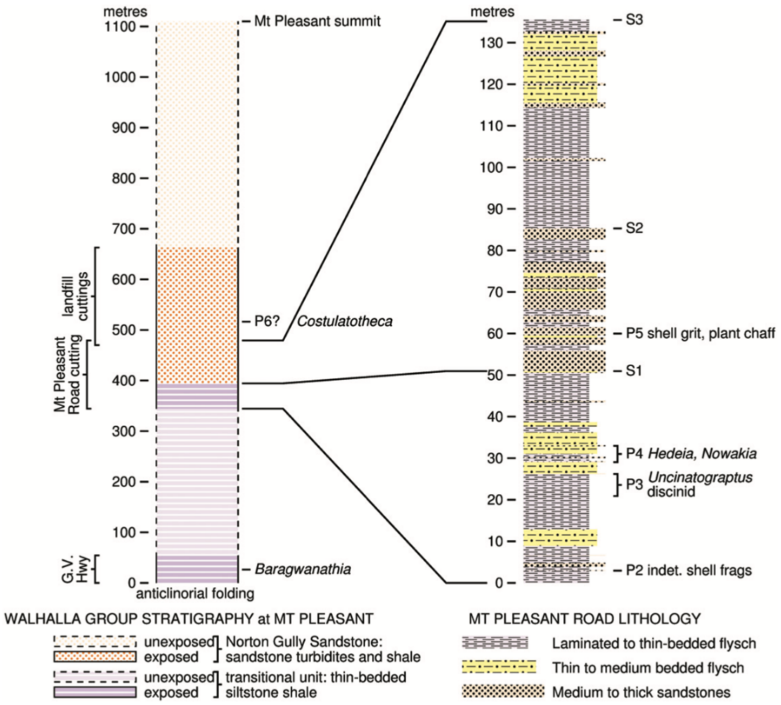

Supplementary figure 1. Stratigraphy and lithology of Mt Pleasant[4], where the studied Baragwanathia flora occurs. From ref. [4].

The exceptional preservation of the plant macrofossils has been interpreted as flysch deposition in a very-low-energy depositional environment with occasional bursts of high-energy turbidites carrying allochthonous fossils from shallower waters[4]. At Mt Pleasant, the plant fragments are smaller than for example the Late Silurian plant assemblage from Limestone Rd near Yea. The taphonomy is consistent with abrupt transport from the place where the plants grew to the site where they were buried. Thus, we rule out a marine origin of the macroplant community, as considered for a related plant species, *Baragwanathia brevifolia*, in the Barrandian area, Czech Republic[6].

S1.2 Sample characterization

The plant fossils are preserved as incrustations in fine-grained sandstone, also recognized as thin to medium bedded flysch[4,5]. The plant itself is now covered in a brown mineral matrix with some organic matter preserved (see below, S1.3). The preservation of the brown material distinguishes the Mt Pleasant fossils from other fossils that we inspected from the Limestone Rd and Frenchman Spur localities that were impression fossils[2,5]. Twenty-five fragments with terrestrial plant remains in four rock specimens were analyzed 41 times. All fossils of taxonomic value, i.e. type specimens, and samples featured on figures and plates in previous publications[5] were avoided (Supplementary figs. 2–5).

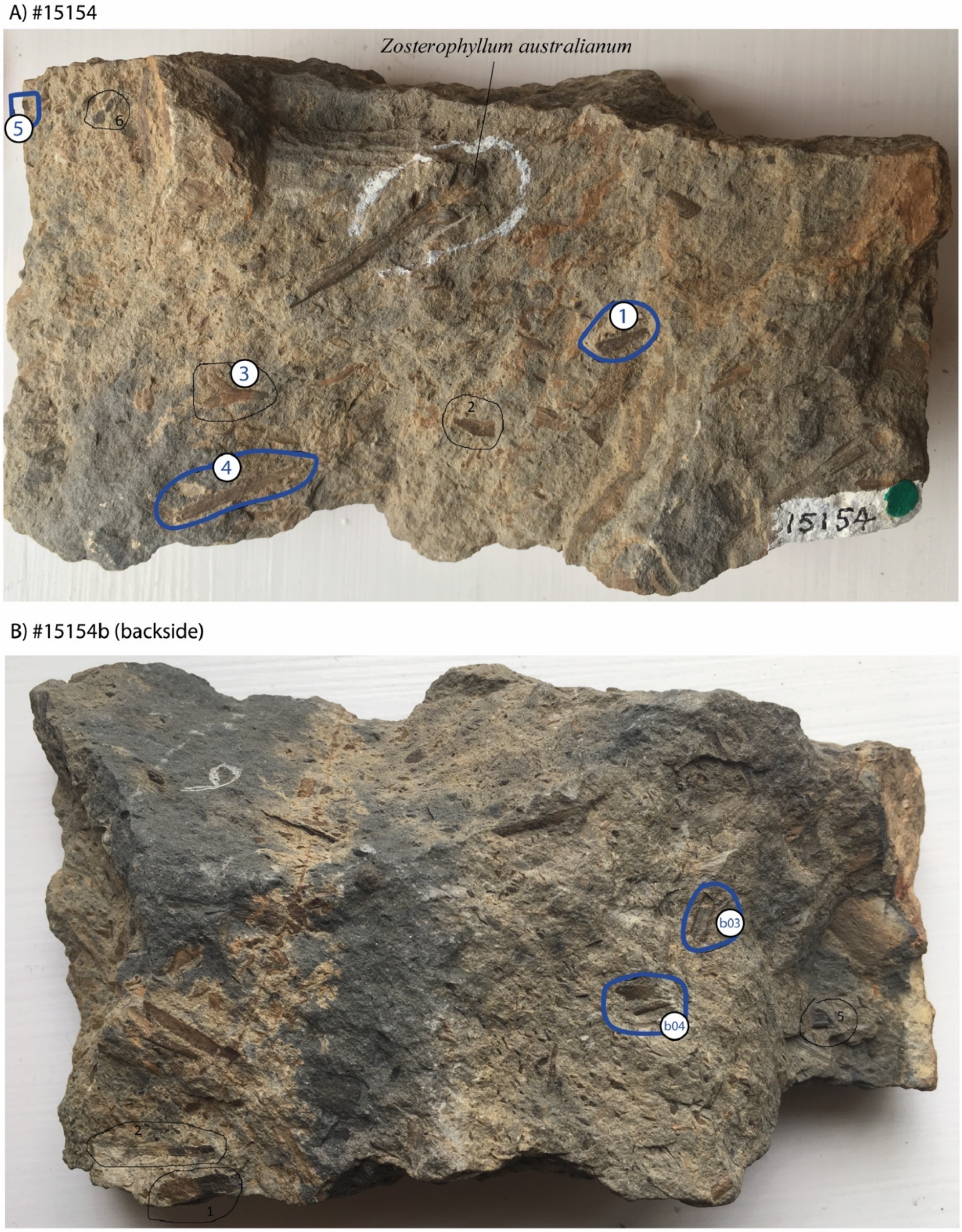

Supplementary figure 2. Sample #15154 and names of fragments studied a) front side and b) backside. Analytical data is summarized in Supplementary Table 1.

A) #15173

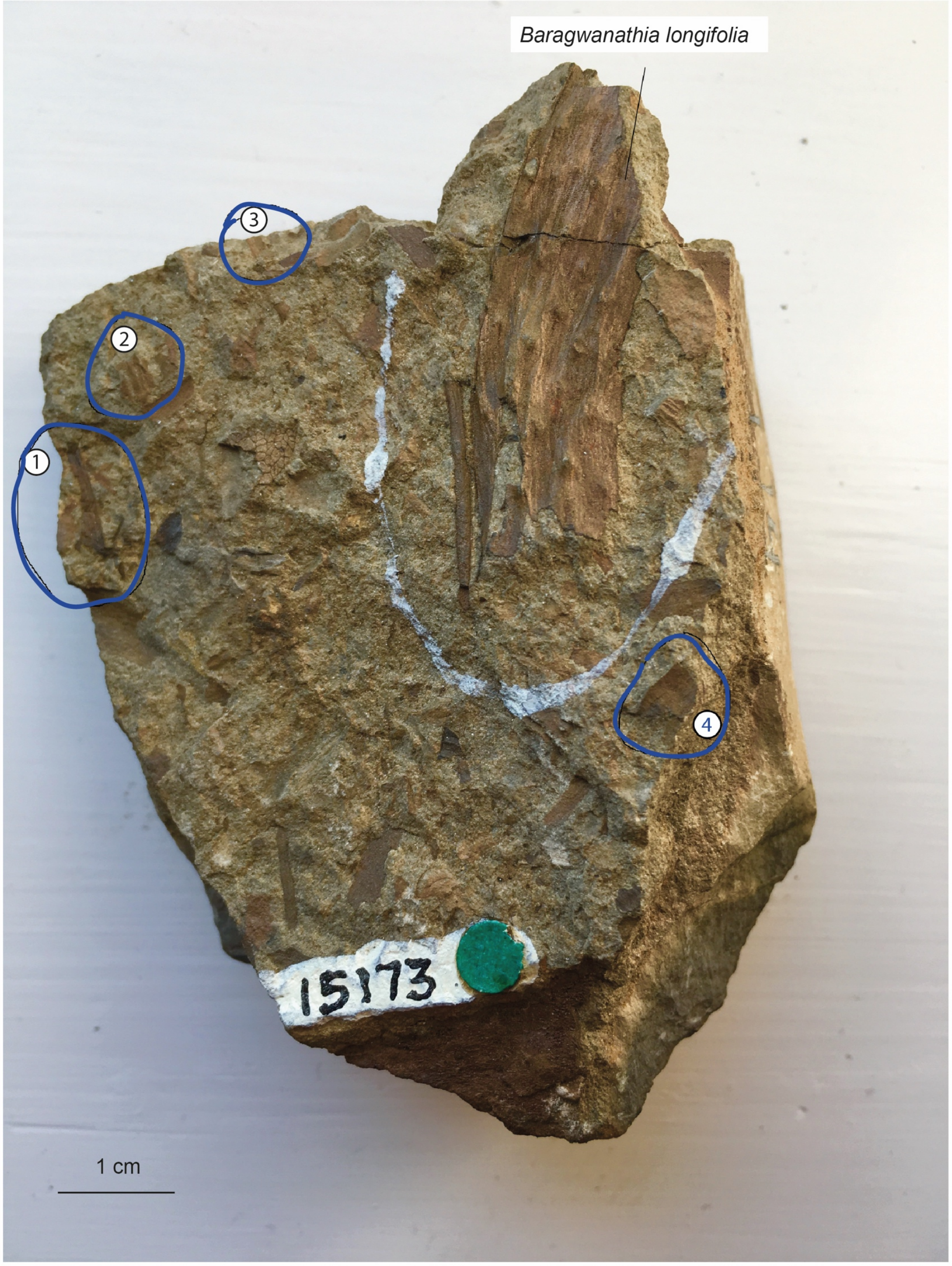

Supplementary figure 3. Sample #15173 with the numbers representing the studied fragments. Analytical data is summarized in Supplementary table 1.

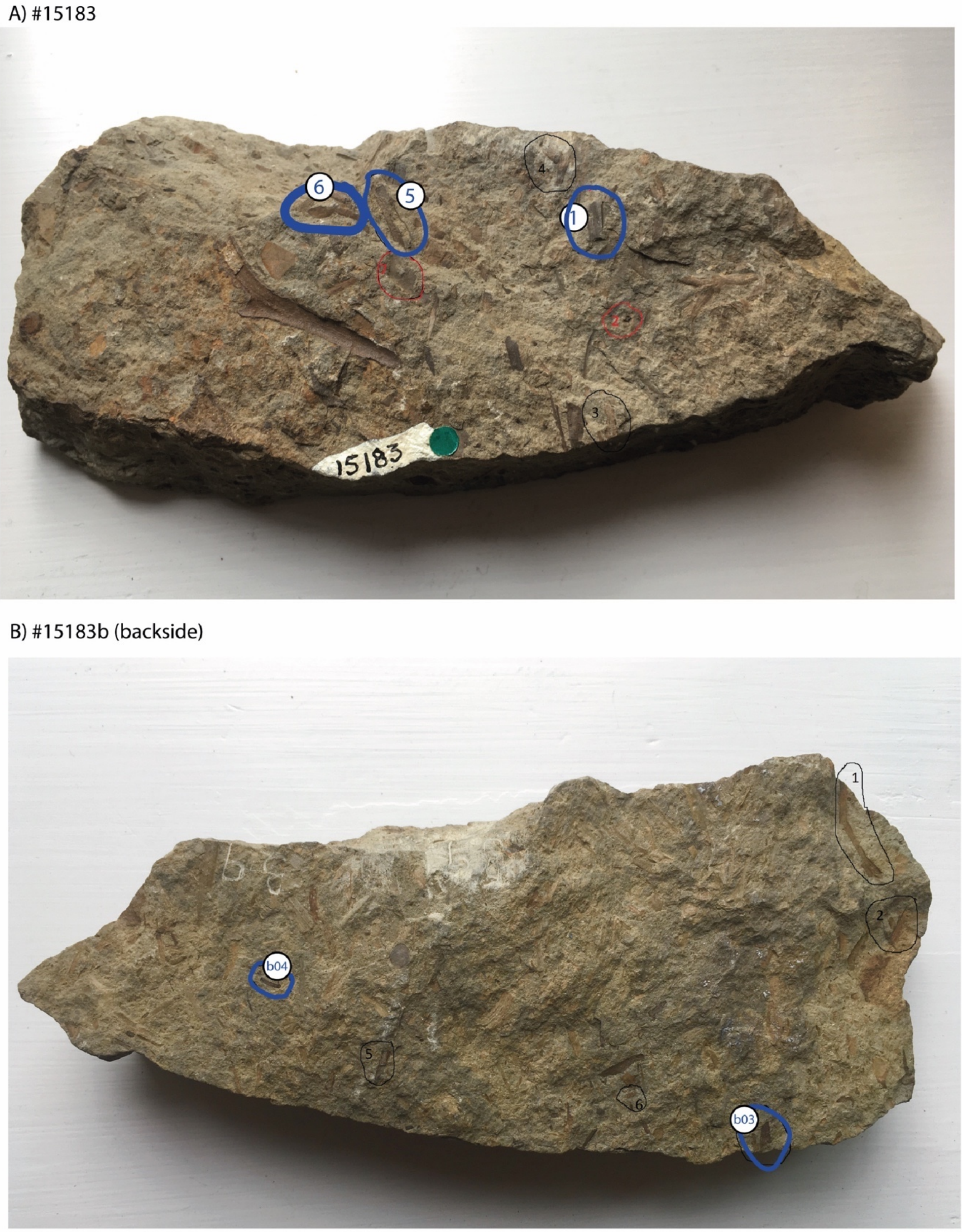

Supplementary figure 4. Sample #15183 and names of fragments studied, a) front side and b) backside. Analytical data is summarized in Supplementary table 1.

S1.3 Characterization of the fossilized plant tissue

S1.3.1 Carbon compounds

Time-of-Flight Secondary Ion Mass Spectrometry (TOF-SIMS) was used to produce semi-quantitative maps of elemental composition in one representative fossil fragment from sample #15153. Supplementary figure 5 summarizes the main results, where calcium (Ca) intensity follows aluminum (Al), whereas carbon-nitrogen (CN^-) follows that of phosphorous (PO_3^-). These data provide clear evidence that carbon is present in the brown mineral matrix, and that C is mainly hosted in an organic phase dispersed over the surface of the fossil, and not in inorganic form; i.e. carbonates. Therefore, we expect that the isotope composition of organic carbon in the fossil remains is derived from the Devonian plant and is not delivered from elsewhere.

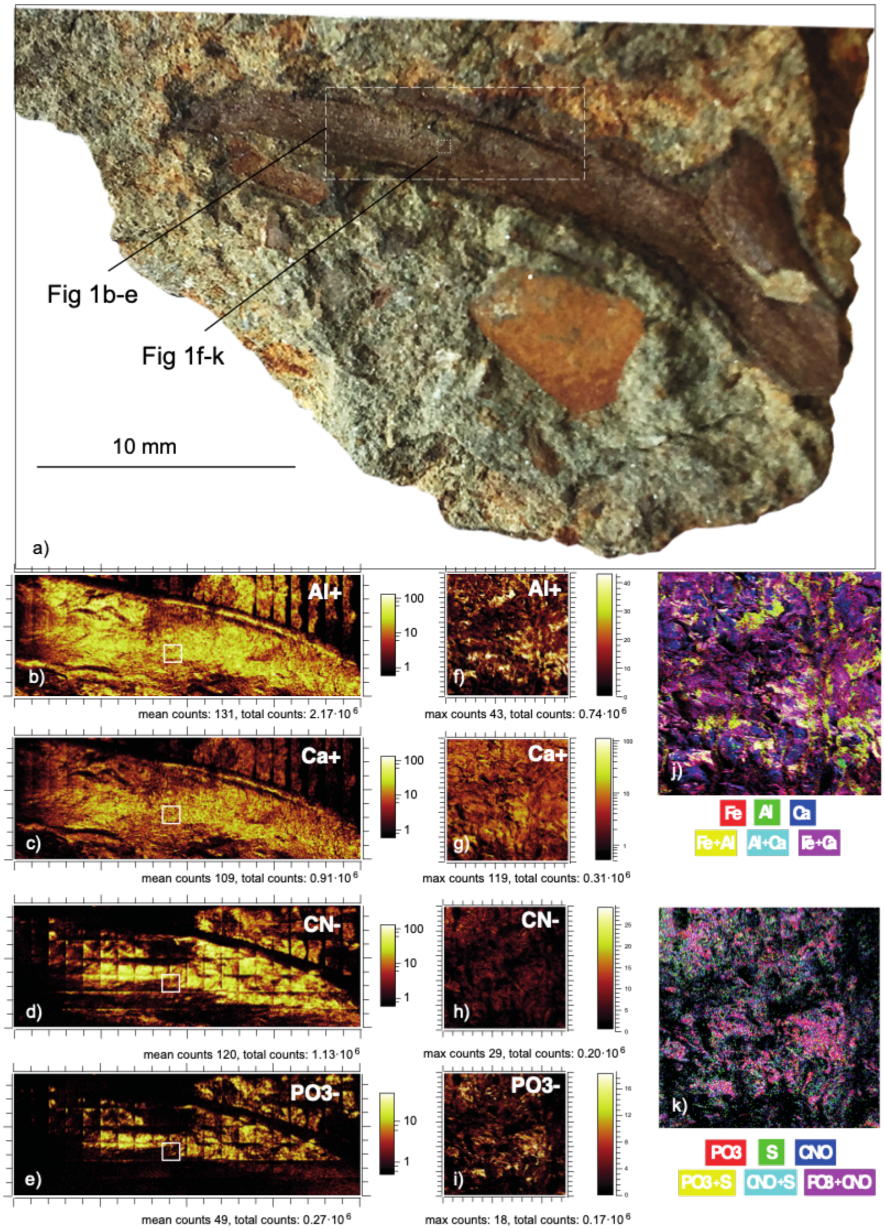

Supplementary figure 5. TOF-SIMS characterization of #15184. a) Optical image. Zooms b-e) and f-k) are 10 and 0.5 mm wide, respectively.

S1.3.2 Carbon isotopes

The carbon isotope composition was measured in 25 fragments of fossilized organic plant tissue (Supplementary table 1). Repeated analyses were made on 14 of these fragments and two fragments were analyzed three times. The $\delta^{13}C_{plant}$ data from fossils fits with a normal distribution (Darling-Anderson criteria) with an average value of 26.7±1.2‰ (1SD, n =24). The variance is larger than analytical uncertainty of the analysis estimated from the reproducibility of standard reference materials (<0.3‰). The isotope variability is real in the sense that variation between distinct plant fragment is also greater than the variance during repeated analyses of the same fragments (except in #15183_01). Thus, we interpret the isotope variation as representative of carbon isotope fractionation in the *Baragwanathia* flora.

NMV #	Material analyzed	$\delta^{13}C$ (V-PDB)
**#15154**	Branched stems	
Fragment 01		-24.4‰
Fragment 02		-27.6‰
Fragment 03		-27.4‰, -27.2‰
Fragment 04		-25.6‰
Fragment 05		-27.9‰, -26.0‰
Fragment 06		-26.7‰, -25.1‰
Fragment b03		-25.2‰, -25.6‰
Fragment b04		-29.8‰
Fragment b05		-25.1‰
**#15173**	Branched stems	
Fragment 02		-28.3‰, -28.2‰
Fragment 04		-28.5‰
**#15174**	Branched stems	
Fragment 01		-26.6‰, -26.8‰
Fragment b02		-27.7‰
Fragment b03		-25.6‰, -24.6‰
Fragment b04		-26.3‰
**#15183**	Branched stems	

Fragment 01		-24.5‰, -27.9‰, -27.6‰
Fragment 04		-27.6‰
Fragment 05		-28.2‰, -28.2‰
Fragment 06		-27.1‰, -27.4‰
Fragment 08		-26.6‰, -27.3‰
Fragment b01		-26.6‰
Fragment b03		-27.0‰
Fragment b04		-26.2‰, -26.6‰, -25.7‰
Fragment b05		-25.2‰, -25.5‰
**#15184**	Branched stems	
Fragment 02		-26.4‰, -26.3‰
All analyses, mean ± SD (n)		-26.68 ± 1.24‰ (n = 41)
All fragments, mean ± SD (n)		-26.75 ± 1.24‰ (n = 25)
**Mean fragments ± SE**		**-26.75 ± 0.25‰**
**Reference materials**		
ANU Sucrose		-10.5±0.3 (n= 3, 1SD)
Peach leaves		-26.0±0.1 (n= 10, 1SD)
USGS 61 (certified -35.05‰)*		-35.10 ± 0.56 (n= 4, 1SD)
USGS 62 (certified -14.79‰)*		-14.81 ± 0.35 (n= 4, 1SD)

Supplementary table 1. Carbon isotope data from the Yea fossil flora. Sample #15154 contain Zosterophyll *australianum*, and #15173 contain fossils described as *Baragwanathia longifolia*. The taxonomy of the studied plant fragments. *run at 30–90 nanomoles of C.

Section S2. Carbon isotope fractionation, stomata size and density in terrestrial plants versus ambient CO_2 level

Terrestrial plants fractionate carbon isotopes during CO_2 uptake mainly due to carboxylation by the Rubisco enzyme and because of diffusion into the cell. The net isotope fractionation (Δ_{leaf}) between plant tissue (δ_p) and ambient air (δ_a) is given as $\Delta = (\delta_a - \delta_p)/(1 + \delta_p)$. The fractionation depends on the water conductance and CO_2 assimilation rate that, in turn, depends on the size and density of stomata on the leaves. Below we calibrate the model in modern lycophytes that are physiologically similar to Devonian shrub-like lycophytes.

S2.1 The gas-leaf exchange model – constraining CO2 assimilation rate and stomatal conductance in the Baragwanathia flora from observed C isotope fractionation, stomata density and stomatal pore size.

Franks (2014)[7] presented a model that derives

- atmospheric pCO_2 (c_a)
- CO_2 assimilation rate (A_n)
- Total conductance (g_{ctot})

from the following observations made in the fossil record:

- C isotope fractionation (Δ_{leaf})
- Stomatal density (SD)
- Stomatal pore length (p)

The solution is found by solving a coupled system of three non-linear equations:

equation 1: $$A_n = g_{c(tot)} \cdot (1 - c_i/c_a) \cdot c_a$$

equation 2: $$A_n \approx A_0 \cdot \frac{(c_a - \Gamma^*)(c_{a0} + 2\Gamma^*)}{(c_a + 2\Gamma^*)(c_{a0} - \Gamma^*)}$$

equation 3: $$\frac{1}{g_{c(tot)}} = \frac{1}{g_{cb}} + \frac{1}{g_{c(op)}} + \frac{1}{g_m}$$

Equation 3 is the equation for hypostomatous microphyll leaves that applies to both fossil lycophytes and modern Huperzias[7]; g_m is the mesophyll conductance determined by an empirical fit: $g_m = (0.0099 \cdot A_n)^{1.0965}$ (ref. *6*) or alternatively, $g_m = 0.013 \cdot A_n$ (ref. *5*), eq. S1.

The total stomata conductance, $g_{c(tot)}$ (mol m^{-2} s^{-1}) is a function of leaf boundary layer conductance to CO_2 (g_{cb}), the operational conductance, $g_{c(op)}$, and mesophyll conductance, g_m. Here, the operational stomata conductance efficiency is to a good approximation a fixed proportion ($\zeta \approx 20\%$) of the maximal conductance (full daylight) and no conductance (night time), $g_{c(op)} = \zeta \cdot g_{c(max)}$. The maximal stomatal conductance, $g_{c(max)}$, is geometrically constrained by the stomatal density (SD), CO_2 diffusivity in air (d), molar volume of air (v) and the stomatal area (a_{max}) and pore depth (l), as:

$$g_{c(max)} \approx \frac{\frac{d}{v} \cdot SD \cdot a_{max}}{l + \frac{\pi}{2}\sqrt{a_{max}/\pi}}$$

The stomatal area is given by $a_{max} = \beta \cdot (\pi p^2/4)$, where β is a shape factor of stomata. A summary of model parameters is given in Supplementary table 3.

S2.2. Numerical solution to the mathematical problem

The system of non-linear equations was solved numerically using Matlab's *fsolve* function. To ensure numerical iterations converged at the right solution, the maximal number of iterations and maximal function evaluations was set to $5 \cdot 10^4$ and $5 \cdot 10^6$, respectively. Solutions were accepted only if the solver reached a solution with small first order optimality (exitflag =1). Convergence was typically achieved in 71% of the random combinations of observables being tested (fig. 2, fig. S8). Uncertainties of observables (Δ_{leaf}, SD, p) were propagated using Monte Carlo sampling of the probability distributions of the observables to the fitted variables (pCO_2, A_n, $g_{c(tot)}$).

S2.3 Calibration in extant lycophytes

S2.3.1 Calibration to Huperzias

To calibrate the model, we investigated two lycophytes species, *Huperzia Squarrosa* and *Huperzia Phlegmaria*, grown in the greenhouse of the Botanical Garden of Copenhagen in natural light at a high relative humidity of 80% and temperature of 19-21°C (night-day) regulated with a thermostat. Both lycophyte species can grow epiphytically and within a soil substrate. Our calibration was performed on plants were grown in soil with six watering periods per day. Peak temperatures in the summer time could reach 28°C. The atmospheric CO_2 level inside the greenhouse was measured overnight on April 20, 2021 using a K30 CO_2 sensor from CO2meter.com. The sensor was calibrated in pure N_2 gas (99.999% N_2 gas, set to 0 ppm CO_2) and set to Copenhagen air (set to 419 ppm).

The atmospheric CO_2 concentration in the greenhouse varied around a daily average of 448±51 ppm with lower concentrations during daytime 412 ppm, when CO_2 assimilation was highest, and 484 ppm during the night, when respiration dominated. Therefore, the modern lycophytes serve as calibration targets for the palaeo-pCO_2-proxy with precision no better than ±10%.

The model depends on the CO_2 assimilation rate (A_0) at standard pressure, and we calibrate this for modern lycophytes grown under optimal relative humidity (=RH ~80%) and c_{a0} = 448±51 ppm. Supplementary table 2 shows the stomatal density and size in two species of *Huperzias* with the measured C isotope fractionation (Δ_{leaf}) reported relative to ambient air with $\delta^{13}C$ of -8.5‰. Using the average values and observed uncertainties, we used the gas-exchange model with the observed ambient pCO_2 level in the greenhouse to calculate A_0 values of 2.2 and 3.5 for *H. squarrosa* and *H. phlegmaria,* respectively. These values are indistinguishable from that found for other lycophytes, and the average of six distinct species is A_0 =3.7±1.6 (1SD), incl.

Diphasiastrum digitatum, Lycopodium annotinum, *Lycopodium clavatum, Lycopodium obscurum, Selaginella longipinnae, Selaginella pallescens*[9].

Using the average A_0 value for modern lycophytes, we calculate the partial pressure of CO_2 in the greenhouse to 561 ± 100 and 413 ± 51 ppm from observations of *H. Squarosa* and *H. phlegmaria*, respectively. This means that the "all-lycophyte calibration" of the leaf-gas exchange model reproduced ambient CO_2 level for our modern lycophyte cultures (448±51ppm) to within an error of approximately ±100 ppm. Therefore, we should not interpret smaller variations than this when reconstructing past pCO_2 from lycophyte fossils.

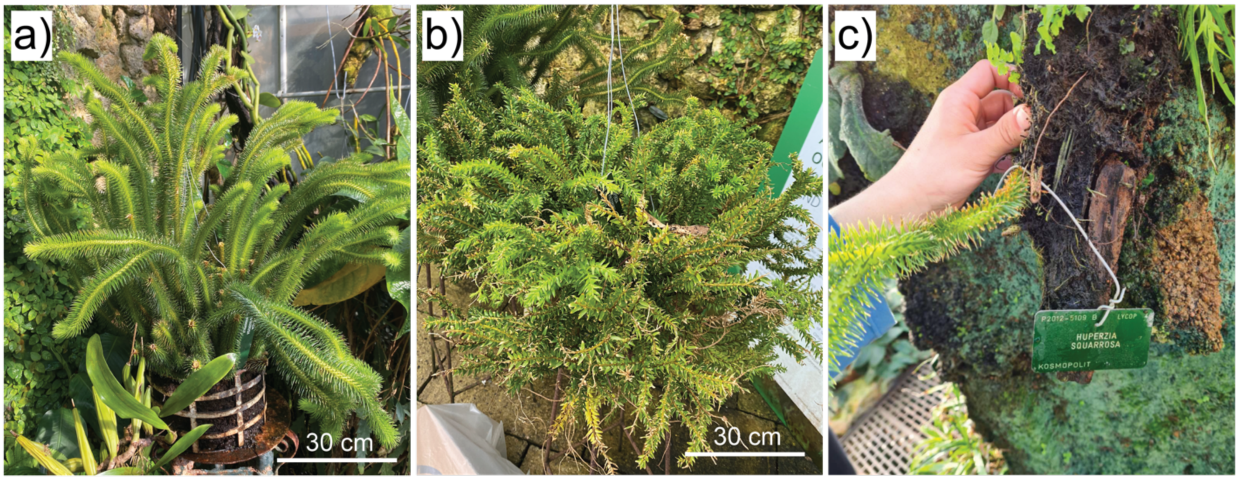

Supplementary figure 6. Modern lycophytes a) *H. squarrosa* and b) *H. phlegmaria* both grown at high relative humidity (RH ~ 80%) and c) *H. squarrosa* grown semi-epiphytically at lower humidity (RH ~60%) in the Botanical Garden, Natural History Museum of Denmark, University of Copenhagen.

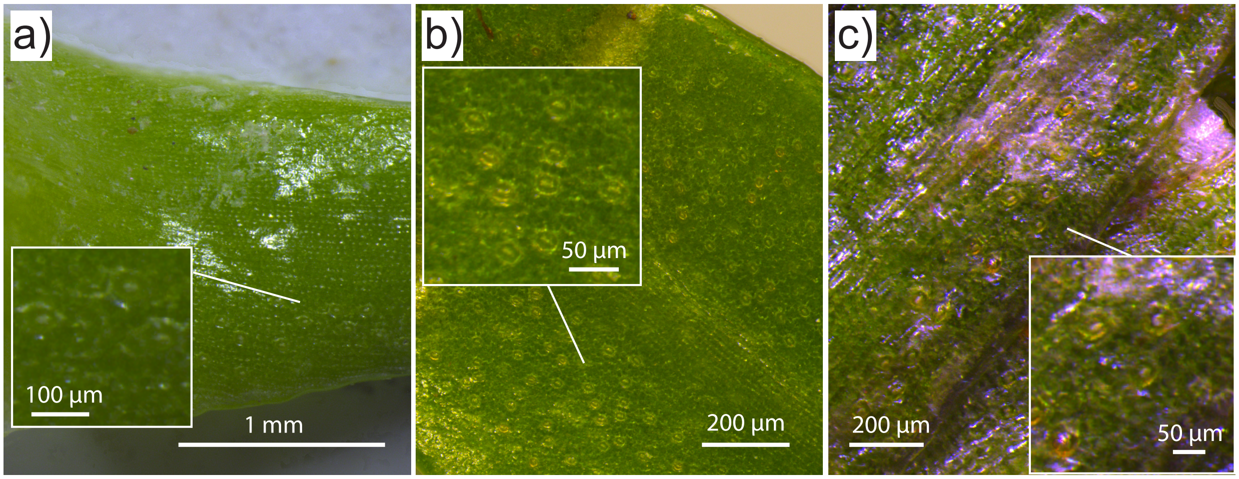

Supplementary figure 7. Stomata on modern lycophytes a) *H. squarrosa* and b) *H. phlegmaria* both grown at high relative humidity (RH ~ 80%) and c) *H. squarrosa* grown semi-epiphytically at lower humidity (RH ~60%) in the Botanical Garden, Natural History Museum of Denmark, University of

Copenhagen. A total of 154 and 190 stomata was counted on 8.60 mm^2 and 8.47 mm^2 of two microphyll leaves from *H. squarrosa*, corresponding to SD = 18-22 mm^{-2}, respectively. The stomata pore length was measured on a subset of well-exposed stomata to 23.7±4.9 µm (1 std. dev., n = 57) and 23.4±3.9 µm (1 std. dev., n =60), respectively. A complete list with all stomata data and SEM photos of the studied modern lycophytes are stored in the Electronic Research Data Archive. NB! The stomata are only present on the abaxial side and are not evenly distributed on the microphyll. The insets in a, b and c show areas with higher SD and slightly smaller p than the average of larger leaf areas.

Species	Stomatal density, SD (mm-2)	Stomatal pore length, p (µm)	Δ_{leaf} (‰)
***H. phlegmaria*, RH~80%**			
– leaf #1	30.35	20.8±3.2	
– leaf #2	27.05	18.6±2.4	
– leaf CM7			19.4
**average ± 1SD**	**28.7±2.3**	**19.7±1.6**	**19.4**
***H. squarrosa*, RH~80%**			
– leaf #2	22.1	23.7±4.9	
– leaf #4	18.2	23.4±3.9	
– leaf #CM6			21.3
– leaf #CM6, rpt			20.8
– leaf #CM6, rpt2			20.2
– leaf #CM6, rpt3			20.5
**average ± 1SD**	**20.1±2.7**	**23.5±4.4**	**20.7±0.5**
***H. squarrosa*, RH~60% (semi-epiphytic)**			
– leaf #1	17.6	22.3±4.2	
– leaf #2	10.8	24.0±3.0	
– leaf #3	19.8	22.1±3.3	
– leaf #CM1			13.1
– leaf #CM5			13.4
**average ± 1SD**	**16.1±4.7**	**22.9±1.0**	**13.3±0.2**

Supplementary table 2. Observed stomatal density, pore size and carbon isotope fractionation in two species of modern lycophytes (*H. squarrosa* and *H. phlegmaria*) grown under optimal conditions in the Botanical Garden at University of Copenhagen, Denmark. The average ambient pCO_2 was measured to 448 ppm, RH – Relative Humidity, rpt – repeated analysis.

S2.4 Application to Devonian lycophytes

The atmospheric pCO_2 estimates from lycophyte fossils are derived from 4 observables (p, SD, $\Delta^{13}C_{leaf,}$ where $\Delta^{13}C_{leaf}$ derived from $\delta^{13}C_p$ and $\delta^{13}C_{air}$) and 11 model constants (A_0, g_{cb}, ζ, l/p, Γ^*, β, c_{a0}, a, b, d, v). A summary of the results from the 10 fossil locations is listed in Supplementary table 3, and a complete list with all model input and model outputs is found in Supplementary Data 1. The errors associated with the observables are propagated using a Monte Carlo approach as exemplified below, while keeping the 11 model parameters constant at their best estimated

value (Supplementary table 4). The sensitivity of the pCO_2 estimates to the choice of model constants is explored in section S2.5.

FA	Fossils	Age Ma	p µm	SD mm^{-2}	$\delta^{13}C_{leaf}$	$\delta^{13}C_{air}$	$\Delta^{13}C_{leaf}$	pCO_2 ppm
1	D	380.46	17.0±3.0	20.8±2.6	-26.5±1.0	-8.1±0.5	18.4±1.1	$581^{+79/-64}$
2	D	387.85	17.0±3.0	17.0±2.0	-24.9±0.6	-5.9±0.5	19.0±0.8	$698^{+100/-77}$
3	D	387.85	17.0±3.0	17.0±2.0	-23.3±1.0	-5.9±0.5	17.4±1.1	$607^{+82/-64}$
4	D	394.05	17.0±3.0	18.0±4.0	-24.1±1.0	-7.5±0.5	16.6±1.1	$552^{+90/-82}$
5	D	401.58	17.0±3.0	16.6±2.0	-25.5±1.0	-8.2±0.5	17.3±1.1	$610^{+83/-68}$
6	D	402.33	17.0±3.0	16.6±2.0	-26.7±0.9	-8.2±0.5	18.5±1.0	$679^{+101/-80}$
7	D	404.59	17.0±3.0	16.6±2.0	-25.6±0.9	-7.8±0.5	17.8±1.0	$639^{+89/-69}$
8	D	408.36	17.0±3.0	18.0±4.0	-26.0±1.0	-6.4±0.5	19.6±1.1	$714^{+144/-102}$
9	B	409.10	28.0±5.0	20.0±3.0	-26.8±1.3	-6.4±0.5	20.3±1.3	$533^{+77/-76}$
10	A	409.10	19.0±1.0	21.5±10.0	-24.9±0.5	-6.4±0.5	18.5±0.7	$516^{+136/-95}$

Supplementary table 3. A summary of the atmospheric pCO_2 results obtained from the 10 Devonian sites. Errors ranges (median ± quartiles) were estimated by Monte Carlo sampling the uncertainty associated with the observables (p, SD and $\Delta^{13}C_{leaf}$) given by normal distributions with the average and standard deviation reported here. All other model inputs are kept constant at their best estimated values: A_0 = 3.73 µmol m^{-2} s^{-1}, g_{cb} = 2.0 mol m^{-2} s^{-1}, ζ = 0.20, l/p = 0.4, Γ^* = 40 µmol/mol, β = 0.6, c_{a0} = 448 ppm, a = 4.4‰, b = 30‰, d = $16 \cdot 10^{-6}$ m^2 s^{-1}, v = 22.4 L/mol. See Supplementary Data 1 for complete details and references to locations, fossil species, isotope data, as well as other output parameters (incl. A_n, g_{tot}, a_{max}, g_{cmax}, c_i/c_a). Abbreviations: A = *Asteroxylon,* B = *Baragwanathia*, D = *Drepanophycus*. FA – Fossil assemblage #.

Asteroxylon, Rhynie Chert, Scotland (FA 10)

A Pragian (~409 Ma) atmospheric pCO_2 estimate of $525^{+139/-101}$ ppm is derived from *Asteroxylon mackiei* in the Rhynie Chert, Scotland. Stomata data has been reported from three specimens (p = 19.0±1.0 µm, SD = 21.5±10 mm^{-2})[10]. Carbon isotope analyses of plant tissue comes from four analyses ($\delta^{13}C_{leaf}$ = –24.9±0.5‰)[11], the carbon isotope composition of the Pragian atmosphere ($\delta^{13}C_{air}$ = –6.4±0.5‰) is derived from numerous analyses of the $\delta^{13}C$ composition of seawater from four different locations (Supplementary table 5). Together, this leads to a carbon isotope fractionation in the Devonian plant tissue relative to ambient air of Δ_{leaf} =18.5±0.7‰.

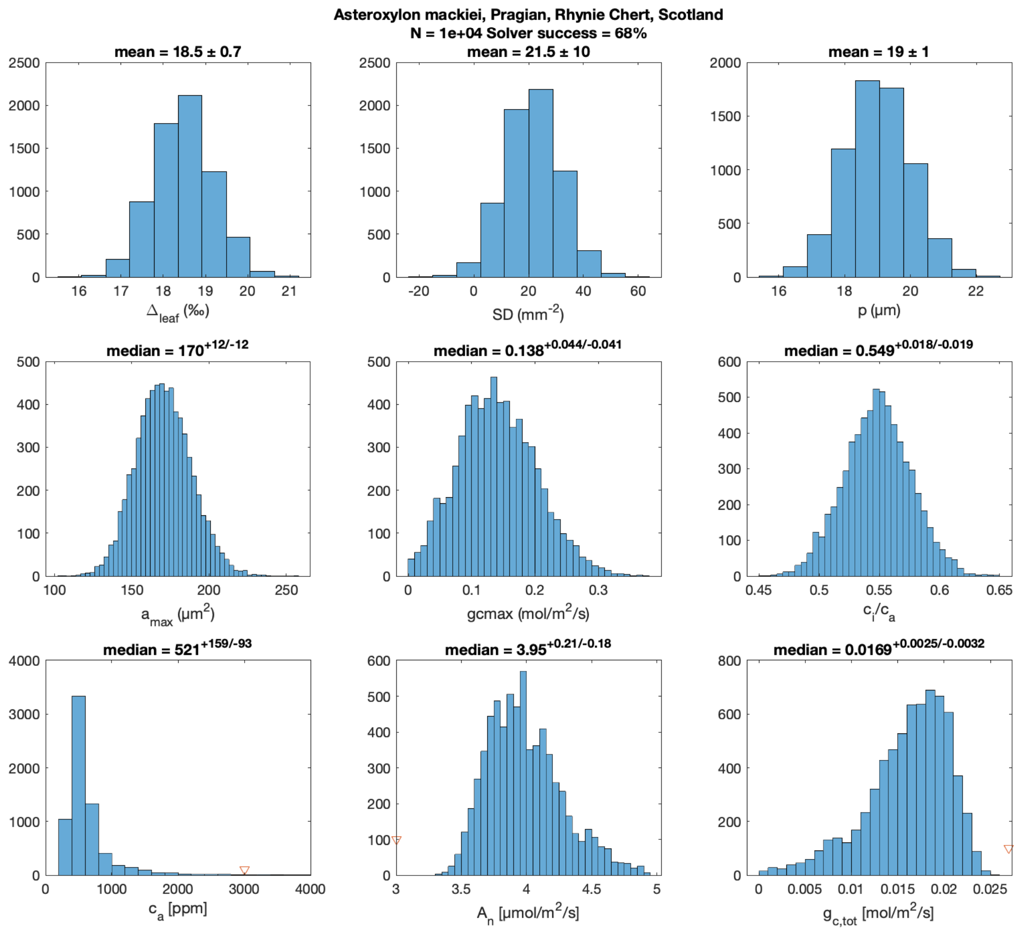

Supplementary figure 8. Error propagation of the mechanistic leaf gas-exchange pCO_2 proxy. Model input parameters are sampled from (N =10,000) from probability distributions for observational data (Δ_{leaf} , SD, p; upper row) (Supplementary Data 1; summarized in Supplementary table 6 and in the supplementary text above) to calculate probability distributions for model parameters including (middle row): stomata area, a_{max}, the maximal conductance at full daylight, $g_{c,max}$, and ratio of CO_2 concentration in the substomatal cavities to atmospheric CO_2, c_i/c_a and (lower row) atmospheric CO_2, c_a, CO_2 assimilation rate A_n, and total conductance, $g_{c,tot}$ for the Pragian lycophyte *Asteroxylon mackiei* from the Rhynie Chert in Scotland. Solutions to the three coupled non-linear equations were obtained with Matlabs *fsolve* function. First-order optimal solutions (misfit converging to zero as iterations proceeds) were obtained in 71% of the iterations with a maximum of $5 \cdot 10^6$ function evaluations (options.MaxFunEvals = $5 \cdot 10^6$). The initial guess for the output parameters are marked with red triangles, and the results do not depend on the initial guess. The median values ± quartiles are reported above each panel. A similar figure is given for the Emsian *Drepanophycus spinaeformis* in fig. 2.

Baragwanathia, Victoria Australia (FA 9)

A Pragian (~409 Ma) atmospheric pCO_2 level of $532^{+77/-76}$ ppm based on observations of fossil fragments from the *Baragwanathia* flora. The geological settings and fossil preservation is described in detail in section S1. Carbon isotope fractionation in plant tissue (Δ_{leaf} = 20.1±1.1‰) was obtained $\delta^{13}C$ analyses of 25 plant fragments (Supplementary table 1) and $\delta^{13}C_{air}$ of the Pragian atmosphere (Supplementary table 5). This data is combined with an estimate for the

stomata density (SD = 28±5 mm^{-2}) and stomatal pore length (p = 20±3 μm) in microphyll of coeval *B. abitibiensis* from Ontario[8]. A conservative estimate for the uncertainties was adopted based on 2 s.d. of repeated analyses of modern lycophytes (Supplementary table 2).

The model also constrains the net CO_2 assimilation rate, A_n = 3.60±0.15 μmol m^{-2} s^{-1} and stomatal conductance, g_{ctot} = 0.019±0.002 mol m^{-2} s^{-1} for the *Baragwanathia* flora. Both values are reasonable for lycophytes and lower than that of modern gymnosperms (A_n = 4.5–7.9 μmol m^{-2} s^{-1}, g_{ctot} = 0.026–0.042 mol m^{-2} s^{-1})[7].

***Drepanophycus*, Siegburg, Germany (FA 8)**

A Siegenian (Pragian-Emsian, ~408.36 Ma) atmospheric pCO_2 constraint of $715^{+140/-102}$ ppm is derived based on carbon isotope data of plant tissue in one fossil *Drepanophycus* sp. from the Wahnbachschichten Fm at Munchshecke, Germany[12] and $\delta^{13}C_{air}$ of the Pragian atmosphere (Supplementary table 5). Due to the lack of stomata data from this locality, we adopted the average stomata density and pore length (± 1 standard deviation) from Devonian *Drepanophycus spinaeformis* (6 localities, 58 samples)[10]. An assessment of the potential error induced by using unpaired isotope-stomata data is reported in the main text.

***Drepanophycus*, at Seal Rock Gaspé, Quebec, Canada (FA 7)**

Emsian deposits (~404.59 Ma) from the Battery Fm at Seal Rock in Gaspé, Quebec (Qbc), Canada yields an average atmospheric CO_2 level of $638^{+88/-69}$ ppm, when 11 carbon isotope analyses of plant tissue (plant, stem) from *Drepanophycus sp.* and *Drepanophycus spinaeformis*[12] and the average composition of the coeval Emsian atmosphere ($\delta^{13}C_{air}$ = -7.8±0.5‰)[13] are paired with stomata data from two fossils from the same location[10,14].

***Drepanophycus*, New Brunswick and Gaspé, Quebec Canada (FA 6)**

Emsian fossils (~402.33 Ma) from four close-by localities from the Campbellton Fm and time-equivalent part of Battery Fm[15] yield an average atmospheric CO_2 level of $679^{+104/-80}$ ppm using carbon isotope data from the coeval Emsian atmosphere ($\delta^{13}C_{air}$ = -8.2±0.5‰)[13] and 19 fossil samples (plant, stem). The average carbon isotope composition of plant tissue ($\delta^{13}C_{leaf}$ = -8.2±0.5‰) from several *Drepanophycus* species (*Drepanophycus sp., Drepanophycus spinaeformis, Drepanophycus gaspianus*) from four close-by localities in Gaspé, Qbc and nearby area on the South Shore of Chaleur Bay in New Brunswick (NB), incl. Dalhousie, NB, L'Anse-a-brillant, Gaspé, Qbc, Locality F, NB, and North shore, Gaspé, Qbc. The carbon isotope fractionation in the plant tissue (Δ_{leaf} = 18.5±1.0‰) is combined with stomata data from the nearby and slightly older fossil assemblage at Seal Rock (FA 6). An assessment of the maximal error induced by using unpaired isotope-stomata data is reported in the main text.

***Drepanophycus*, Mosel Valley, Germany (FA 5)**

An Emsian (~401.58 Ma) atmospheric pCO_2 constraint of $613^{+90/-68}$ ppm is derived based on carbon isotope data of plant tissue in one specimen *Drepanophycus spinaeformis* from the Nellenköpfchen Formation at Alken Quarry in Mosel Valley, Germany[12] and $\delta^{13}C_{air}$ of coeval Emsian atmosphere ($\delta^{13}C_{air}$ = -8.2±0.5‰)[13]. Due to the lack of stomata data from this locality, we used the average stomata density and pore length (SD = 16.6±2.0, 1 standard deviation)[10] from coeval Emsian fossils from Seal Rock, Battery Pt in Gaspé Peninsula Qbc and slightly younger close-by localities in Quebec and New Brunswick[14]. An assessment of the potential error induced by using unpaired isotope-stomata data is reported in the main text.

***Drepanophycus*, Maine, USA (FA4)**
A slightly lower atmospheric pCO_2 estimate of $554^{+91/-84}$ ppm compared to earlier Emsian deposits is derived from Emsian-Eifelian (~394.05 Ma) deposits, due to slightly lower Δ_{leaf} = 16.6±1.1‰, in the fossil samples from Trout Valley Formation at Traveler Mountains in Maine, USA. The carbon isotopic composition of the atmospheric at this time is estimated to $\delta^{13}C_{air}$ = –7.5±0.5‰[13], and the carbon isotope composition of the plant tissue ($\delta^{13}C_{leaf}$ = –24.1±1.0‰) has been obtained from stem and leaves in four specimens (3 *Drepanophycus spinaeformis, 1 Drepanophycus sp.*)[12]. Due to the lack of stomata data from this locality, we adopted the average stomata density and pore length (SD = 17.2±4 mm^{-2}, 1 standard deviation) from Devonian *Drepanophycus spinaeformis* (6 localities, 58 samples)[10]. An assessment of the potential error induced by using unpaired isotope-stomata data is reported in the main text.

***Drepanophycus*, Xinjiang, China (FA3)**
A Givetian (~387.85 Ma) atmospheric pCO_2 estimate of $608^{+83/-65}$ ppm is derived from plant tissue from one specimens (*Drepanophycus sp.*)[12] in the Hujiersite Formation at Xinjiang, China. The carbon isotopic composition of the atmosphere at this time is higher than in the older sites, $\delta^{13}C_{air}$ = –5.8±0.5‰[13], and so is the carbon isotope composition of the plant tissue ($\delta^{13}C_{leaf}$ = –23.3±1.0‰). Due to the lack of stomata data from this locality, we used stomatal data from coeval Givetian *Drepanophycus spinaeformis* in New York, USA (FA 2, see below)[10]. An assessment of the potential error induced by using unpaired isotope-stomata data is reported in the main text.

***Drepanophycus*, Hamilton Fm, New York, USA (FA2)**
An atmospheric pCO_2 estimate of $695^{+99/-73}$ ppm is derived from fossil samples in the Givetian (~387.85 Ma) Hamilton Formation at Schoharie County, and Cairo Quarry at Green County, New York, USA. The carbon isotopic composition of the atmosphere at this time is estimated to $\delta^{13}C_{air}$ = –5.8±0.5‰[13], and the carbon isotope composition of plant tissue in two specimens of *Drepanophycus* sp. yields $\delta^{13}C_{leaf}$ = –24.9±1.0‰[12], which means the carbon isotope fractionation in the plant was Δ_{leaf} = 19.0±0.8‰,. The average stomata density (17±2 mm^{-2}) and stomata pore length (17±3 µm) are derived from 17 specimens of *Drepanophycus spinaeformis* from Quarry South, Kiskinton Fm at Cairo and from Panther Mt Fm at Schoharie River, New York[14,10], USA.

***Drepanophycus*, Oneonta Fm New York, USA (FA1)**
A Frasnian (~380.46 Ma) atmospheric pCO_2 estimate of $579^{+75/-63}$ ppm is derived from fossils from the Oneonta Formation at Green County, New York, USA. At this time, the carbon isotope composition of the atmosphere (as derived from the seawater isotope record, $\delta^{13}C_{air}$ = –8.1±0.5‰[13]), and the carbon isotope composition of plant tissue in one *Drepanophycus spinaeformis* specimen ($\delta^{13}C_{leaf}$ = –26.5±1.0‰[12]) are both lower than in the Givetian. Thus, the carbon isotope fractionation induced by the lycophyte, Δ_{leaf} = 18.4±1.1‰, was similar to that of Givetian ancestors. The average stomata density (20.8±2.6 mm^{-2}) and stomata pore length (17±3 µm) are derived from 12 specimens of *Drepanophycus spinaeformis* from two localities near Pratsville, including Cave Mt, Oneonta Fm, Geneseo Group, New York, USA[14,10]. These stomatal data from fossils are paired with isotope data from the nearby location of similar age.

S2.5 Sensitivity analyses

Our palaeo-atmospheric pCO_2 constraints falls with a relatively narrow range of uncertainties, compared to 3-12 times higher pCO_2 estimates previously reported using other approaches (see section S3). To explore the robustness of our new result, we conducted a sensitivity analysis to evaluate the importance of the model input. We distinguish between i) the observables, ii) the model constants, as well as iii) two distinct model parameterizations that utilize distinct empirical relationships between stomatal conductance (g_m) and CO_2 assimilation rate (A_n) [7,16].

i) All errors associated with the observables are propagated in all reported pCO_2 estimates, for example for the Emsian *Drepanophycus case study* with the default parameterization yields an atmospheric pCO_2 level of $529^{+94/-94}$ ppm using stomatal pore length (p = 17±3 µm), stomatal density (SD = 18±4 mm^{-2}) and ^{13}C fractionation by plant relative to ambient air (Δ_{leaf} = 16.1±1.1‰), where the latter is obtained from the carbon isotope composition of plant tissue and ambient air (section S2.4). A smaller total stomata area (lower p and/or lower SD) would yield higher atmospheric pCO_2 (Figure 1). Variable and lower stomata density has been reported[17,10] for 26 fossil fragments from the Emsian *Drepanophycus qujingensis* with values between 3.1 and 20.2 mm^{-2}, (e.g. SD = 11.5±1.5 mm^{-2}, mean ± std.dev). The lower SD alone would yield a higher (+221 ppm) atmospheric pCO_2 estimate of $750^{+127/-102}$ ppm, but because the stomata pores of these fossils are longer (p = 50±7 µm. NB! mean of only six pictured specimens[17]), the model actually predicts a lower (–205 ppm) atmospheric pCO_2 level ($324^{+36/-32}$ ppm) for *Drepanophycus qujingensis*, assuming Δ_{leaf} was the same as in other coeval *Drepanophycus* fossils (isotope have not been measured for *D. qujingensis*). Therefore, we conclude that errors associated with variation in the stomata density and pore length could be significant, but it does not alter the main conclusion in this study.

ii) To explore the importance of the other model input, we calculated the pCO_2 estimate for alternative parameterizations of the gas-exchange model using the Emsian *Drepanophycus spinaeformis* as an example (fig. 2).

Supplementary table 4 summarizes the results of the sensitivity analysis. We highlight three of the 11 model constants that could potentially produce pCO_2 estimates outside the error range defined by the uncertainty of the observables. A systematic bias of the model constants is needed to generate higher atmospheric pCO_2 compatible with previous work, and we explore these options further below. Importantly, we conclude that any reasonable choice of value for the model parameters would not yield 10-fold (4000-6000 ppm)[18–24,25] or even 4-fold higher (~2000 ppm) higher pCO_2 estimate than predicted here[26,27].

Sensitivity analysis of		Unit	Explored values (default = **bold** font)	pCO_2 (ppm)	Deviation pCO_2 (ppm)
**Observables**					
Stomata density	SD	mm^{-2}	11.5±1.5	$750^{+127/-102}$	+221
			**18.0±4.0**	**$529^{+94/-94}$ ppm**	**± 0**
Stomata pore length	p	µm	**17.0±3.0**	**$529^{+94/-94}$ ppm**	**± 0**
			50±7	$296^{+45/-33}$	-233
Isotope fractionation in the plant	Δ	‰	**16.6±1.1**	**$529^{+94/-94}$ ppm**	–195
			21.5±1.5	$334^{+57/-39}$	**± 0**
**Model constants**					
Reference CO_2 assimilation rate at c_{a0}	A_0	$\mu mol\ m^{-2}\ s^{-1}$	2.17	$334^{+57/-39}$	–195
			3.0	$437^{+90/-59}$	–92
			**3.73**	**$529^{+94/-94}$ ppm**	**± 0**
			4.56	$616^{+132/-89}$ ppm	+86
Leaf boundary layer conductance to CO_2	g_{cb}	$mol\ m^{-2}\ s^{-1}$	1.8	$529^{+94/-94}$	– 0.4
			2.0	**$529^{+94/-94}$**	**± 0**
			2.2	$532^{+99/-91}$	+ 3
Operational stomata conductance efficiency ($\zeta = g_{c,op}/g_{c,max}$)	ζ	–	0.08	$1206^{+237/-189}$	+676
			0.14	$701^{+156/-107}$	+172
			**0.20**	**$529^{+94/-94}$**	**± 0**
			0.29	$386^{+74/-53}$	-143
			0.38	$325^{+53/-40}$	-204
Stomatal pore shape factor	β	–	0.2	$1112^{+203/-222}$	+582
			0.4	$652^{+141/-89}$	+123
			**0.6**	**$529^{+94/-94}$**	**± 0**
			0.8	$436^{+100/-62}$	–93
			1.0	$391^{+75/-53}$	–138
Reference *p*CO_2	c_{a0}		410	$533^{+114/-86}$	+3
			**448**	**$529^{+94/-94}$**	**± 0**
Stomatal pore depth/length ratio	*l/p*	*µm/µm*	0.3	$482^{+94/-80}$	–47
			**0.4**	**$529^{+94/-94}$**	**± 0**
			0.5	$569^{+106/-88}$	+40
^{13}C fractionation by diffusion	a	‰	4.1	$537^{+97/-92}$	+8
			**4.4**	**$529^{+94/-94}$**	**± 0**
			4.8	$518^{+92/-88}$	–12
^{13}C fractionation by Rubisco	b	‰	27	$605^{+117/-86}$	+76
			**30**	**$529^{+94/-94}$**	**± 0**
			33	$468^{+98/-74}$	–61
CO_2 compensation point in the absence of dark respiration	Γ*	µmol/mol	20	$522^{+86/-81}$	–7
			**40**	**$529^{+94/-94}$**	**± 0**
			55	$537^{+103/-95}$	+8
Diffusivity of CO_2 in air	d	$m^2\ s^{-1}$	**$16 \cdot 10^{-6}$**		–
Molar volume of air	v	L/mol	**22.4**		–
Mesophyll conductance	g_m	$mol\ m^{-2}\ s^{-1}$	$g_m=(0.0099 \cdot A_n)^{1.0965}$	$553^{+95/-80}$	+24
			**$g_m = 0.013 \cdot A_n$**	**$529^{+94/-94}$**	**± 0**

Supplementary table 4. Sensitivity analyses of the model parameters used to derive atmospheric pCO_2 from the gas-exchange model using the *Emsian Drepanophycus spinaeformis* fossil data as reference ($c_a = 529^{+94/-94}$ ppm) (Fig. 2). The absolute pCO_2 estimates (median) and asymmetric error (±quartiles) are given in each parameterization and the deviation from the reference point is shown in the right-most column. The explored parameter range is discussed in the text and in refs[7,16]. Porter et al.[28] suggested O_2-dependency of the photorespiratory compensations point: $\Gamma^* = 1.78 \cdot (pO_2/21atm\%)$ corresponding to $\Gamma^* = 20 - 55$ µmol/mol for any reasonable atmospheric pO_2 level between 12 and 30 atm%. The error range associated with other fossil locations is described in section S2.6 with results summarized in Supplementary Data 1.

Systematically higher pCO_2 estimates would occur with systematically biased model constants, including a) lower operational stomata conductance efficiency ($\zeta < 0.20$), b) far more elongated stomata pore shape ($\beta < 0.2$) compared to that of modern lycophytes or c) a higher CO_2

assimilation rate at reference CO_2 concentration ($A_0 > 3.5$ µmol m^{-2} s^{-1}) in the ancient flora compared to modern lycophytes. We evaluate these below.

ζ – operational stomata conductance efficiency

A higher atmospheric pCO_2 level would be derived if Devonian lycophytes operated at lower operational conductance efficiency than most plants today. The range of ζ-values (0.14–0.29) recorded for five species of living plants (*Eucalyptus globulus, Acmena graveolens, Populus tremuloides, Picea engelmannii, Quercus gambelii*) yields significant pCO_2 offsets of +172/-143 ppm. There is currently no direct measurements of ζ for lycophytes. Yet, the two species of *Huperzia squarrosa* grown semi-epiphytically under poor humidity conditions (Supplementary table 2) might have operated at a lower stomatal conductance efficiency, since the ambient greenhouse pCO_2 level is underestimated by -128^{+79}_{-67} ppm using the default model parameters. Potentially, a lower operational to maximal stomatal conductance ratio of $\zeta = 0.14$ compared to the same plant species grown under ideal (natural habitat) conditions ($\zeta = 0.20$) would explain this discrepancy. Therefore, far lower ζ-values than observed in modern plants and inferred for lycophytes grown under suboptimal conditions are considered unlikely (e.g. $\zeta < 0.10$ indicative of substantially higher atmospheric pCO_2 estimates; Supplementary table 4).

β – stomata pore shape

A higher atmospheric pCO_2 level would be derived if Devonian lycophytes had far more elongated stomatal pores than modern lycophytes. Recall, β defines[7] the maximal opening of stomata compared to a full circle ($\beta = 1$) with a diameter p (stomata pore length); hence $a_{max} = \beta \cdot \pi \cdot p^2/4$.

Modern lycophyte *H. prolifera*[7] display a β value of 0.6. The shape of stomatal pores is more circular ($\beta = 0.8$) in one Emsian specimen of *Drepanophycus sp.* (calculated from Fig. 9I in ref. [10]) and more elongated in *Asteroxylon* fossils ($\beta = 0.4$)[10]. We assumed a β value of 0.6 for all pCO_2 estimates, acknowledging that our results would be systematically biased by ±100 ppm if the average pore shape of the Devonian lycophytes was at one these extremes (0.4–0.8; Supplementary table 4). Currently, there is no indication that Devonian lycophytes had far more elongated stomata pores than modern lycophytes of fossil specimens analyzed to date.

A_0 – CO_2 assimilation rate at reference CO_2 level, c_{a0}

A higher atmospheric pCO_2 level would be derived if Devonian lycophytes had assimilated CO_2 at a faster rate than modern lycophytes under the same reference CO_2 level. We assume a A_0 value similar to the average value of six species of modern lycophytes, which is also compatible with that inferred from cultures of *H. squarrosa* and *H. phlegmaria* in this study. On the basis that ancestors unlikely would outperform their descendants, we rule out that this factor could have systematically underestimated Devonian atmospheric pCO_2 levels. If anything, Devonian plants might have assimilated slower ($A_0 < 3.7$ µmol m^{-2} s^{-1}) and atmospheric pCO_2 predictions should be accordingly lower (Supplementary table 4).

S2.6. Reconstructing atmospheric pCO_2 from other Devonian lycophytes

Supplementary figure 9 shows a compilation of $\delta^{13}C$ data from Devonian C3 plants (*9*, *10*, this study) and marine carbonates[30]. From this, we established a $\Delta^{13}C_{leaf}$ record from lycophyte-like species in the database, including *Drepanophycus* (3 taxa, n = 40), *Haskinsia* (2 taxa, n = 2), *Leclerqia* (4 taxa, n = 23), *Sawdonia,* (3 taxa, n = 28) and the *Baragwanathia* flora (n = 25).

Most Devonian fossils with carbon isotopic data do not simultaneously preserve stomatal data, but the stomata of *Drepanophycus spinaeformis* from Emsian, Givetian and Frasnian deposits carry stomata with similar size and density as modern plants, including SD = 18±4 mm^{-2} and p = 17±3 µm. Their stomatal anatomy is also similar to *Baragwanathia*[8]. Slightly lower stomatal density is reported from the Emsian *Drepanophycus qujingensis* with 11.5±1.5 mm^{-2} and a pore length of about 17±3 µm (*12*, *13*), but we cannot determine whether or not that reflects growth under higher atmospheric pCO_2 because there is no isotopic data for this taxon.

Low stomatal densities are observed in the Emsian zosterophyll *Sawdonia Ornata* (SD = 4.3±1.9, n = 38; [32] with pore length ~17 µm [10]. There is a decent amount of isotopic data from *Sawdonia* fossils (n = 28) that can be used in combination with the stomatal data to calculate ambient atmospheric pCO_2 if we make assumptions about its CO_2 assimilation rate at standard pressure. We find that at low stomatal density, the atmospheric pCO_2 estimates carry large uncertainties (Supplementary fig. 11).

Little is known about *Leclerqia, Haskinsia* as well as major components of Middle and Upper Devonian vegetation (Cladoxylales and progymnosperms), including the 'leafy' taxa, e.g. *Archaeopteris.* Hence, atmospheric pCO_2 levels 410-380 Ma are best constrained by data from lycophytes, including *Drepanophycus*, Asteroxylon and the *Baragwanathia* flora (Supplementary Data 1).

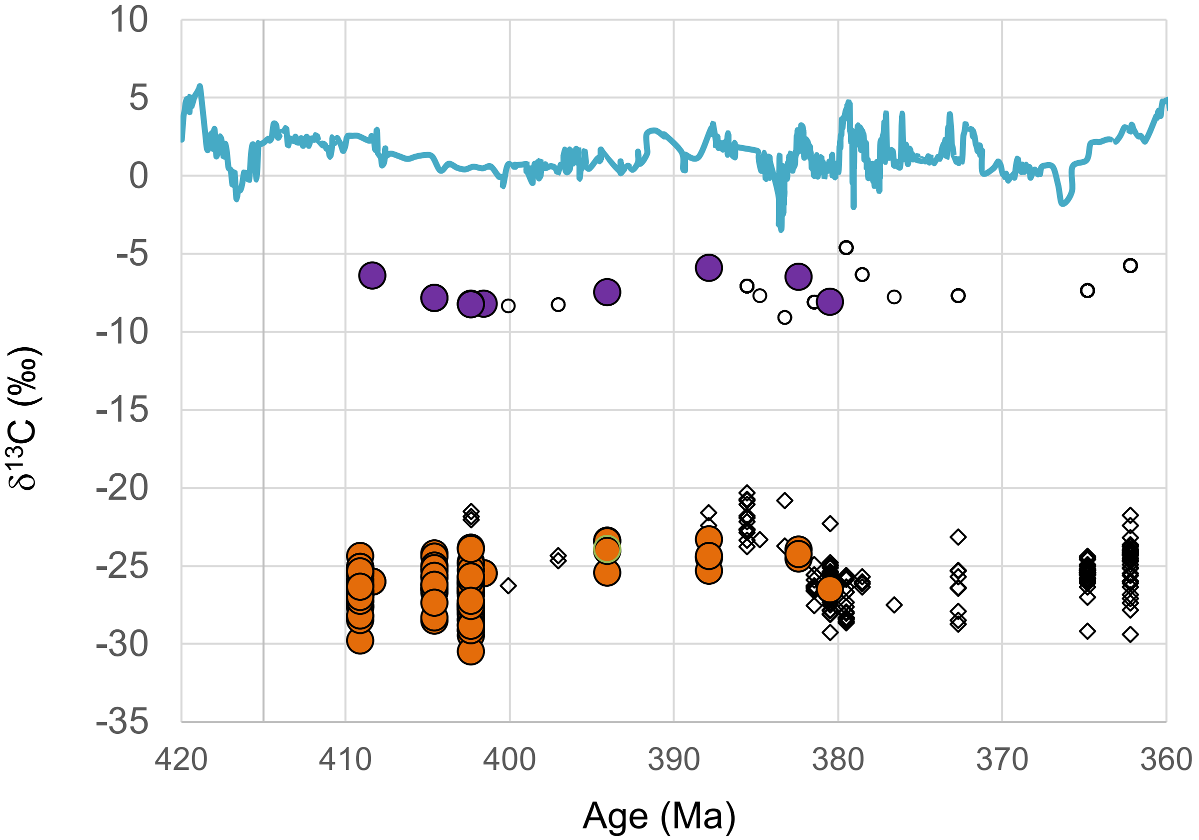

Supplementary figure 9. The carbon isotope composition of marine carbonates (blue curve), air (white circles) and C3 plants (black diamonds). The composition of lycophytes (orange circles) and corresponding air (purple circles) are highlighted. The isotopic composition of air is calculated assuming air-seawater offset of 6.9‰ (Supplementary fig. 13).

The carbon isotope fractionation exerted by Devonian plants is calculated from the isotope composition of terrestrial plants and contemporaneous air (Supplementary fig. 9). The atmospheric CO_2 level was calculated using the approach described in section S2.1 from isotopic and stomatal data of lycophytes with error bars representing Monte-Carlo-propagated analytical uncertainties, 1SD (Supplementary figs. 8 and 11; Supplementary Data 1).

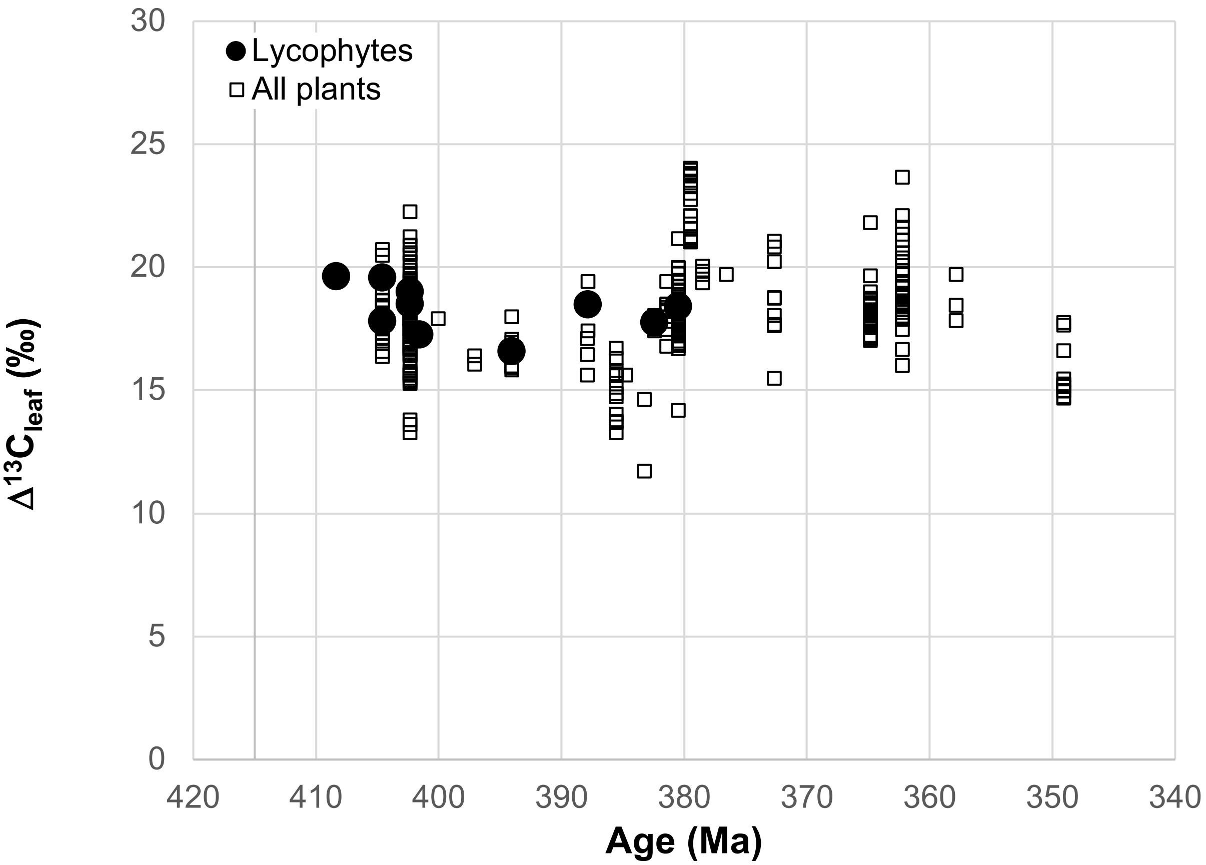

Supplementary figure 10. Carbon isotope fractionation in fossilized C3 plants, $\Delta^{13}C_{leaf}$ (white squares). Lycophytes are highlighted (black circles).

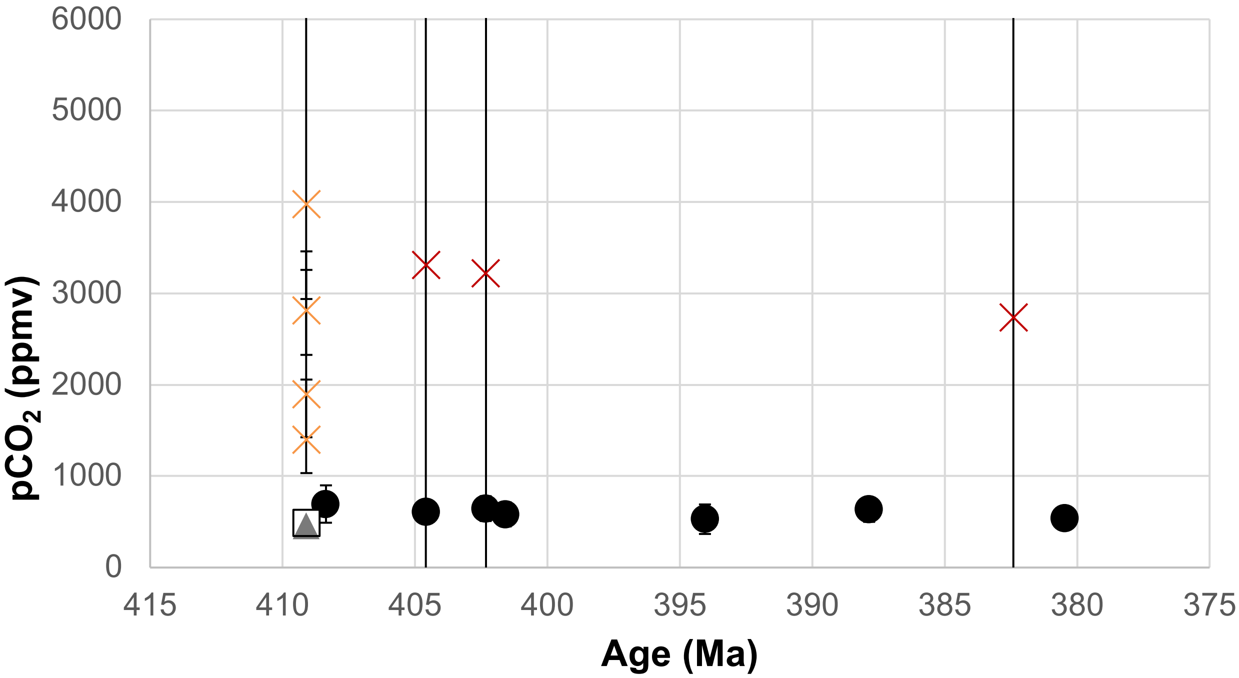

Supplementary figure 11. Atmospheric CO_2 constraints calculated from isotopic and stomatal data from lycophytes – Drepanophycus (●), Asteroxylon (▲) and the Baragwanathia flora (☐), the zosterophyll Sawdonia (x) and rhyniophytes Rhynia, Nothia, Horneophyton, Aglaeophyton (x) using a reference CO_2 assimilation rate of average modern lycophytes, $A_0 = 3.5$ µmol m^{-2} s^{-1}, and an operational stomata conductance efficiency, $\zeta = 0.2$. Error bars represent propagated analytical uncertainties only. The atmospheric pCO_2 constraints derived from extinct zosterophylls and rhyniophytes would yield more precise values consistent with that of lycophytes if their CO_2 assimilation rate was lower A (µmol m^{-2} s^{-1}) than for lycophytes, see main text and Supplementary Data 1.

S2.7 The effect of water utilization efficiency

Spatial Δ_{leaf} variability in land plants arises from variability in the water availability in the environment[33,34]. This is because Δ_{leaf} is strictly a function of the CO_2 concentration in the substomatal cavities (c_i), and c_i depends on the flux of CO_2 into the leaf and CO_2 removal from the leaf by assimilative C fixation. The CO_2 flux into the cell is not only a function of ambient CO_2 levels, but also depends on stomatal conductance into the leaf. At lower water availability, plants down-regulate their stomatal conductance and thereby enhance their Water Utilization Efficiency (WUE), generally resulting in a Δ_{leaf} decrease. Hence, at constant atmospheric pCO_2, Δ_{leaf} is a time-integrated measure of WUE, although leaf temperature and mesophyll conductance are confounding factors. The relative effect of these factors is clear from a comparison of fractionations (Δ_{leaf}) observed in modern C3 plants as a function of mean annual precipitation rate (Supplementary fig. 12). Here, fractionations can be muted by ~4‰ in drier areas, but are invariant at >1000 mm/yr.

Potentially, spatial records of fossilized plant material can be used to constrain water utilization efficiency (WUE) and proximity to aquifers the early flora, as has been suggested for Mid-Late Devonian land plants[12]. Our new data from the *Baragwanathia* flora comes from humid, tropical

settings with mean annual precipitation in excess of 1500 mm/yr according to our paleoclimate models (Section S4). Therefore, a high water-use efficiency for the *Baragwanathia* flora (that are physiologically similar to modern lycophytes) can be ruled out as explanation for muted fractionations observed in the Early Devonian plant fragments.

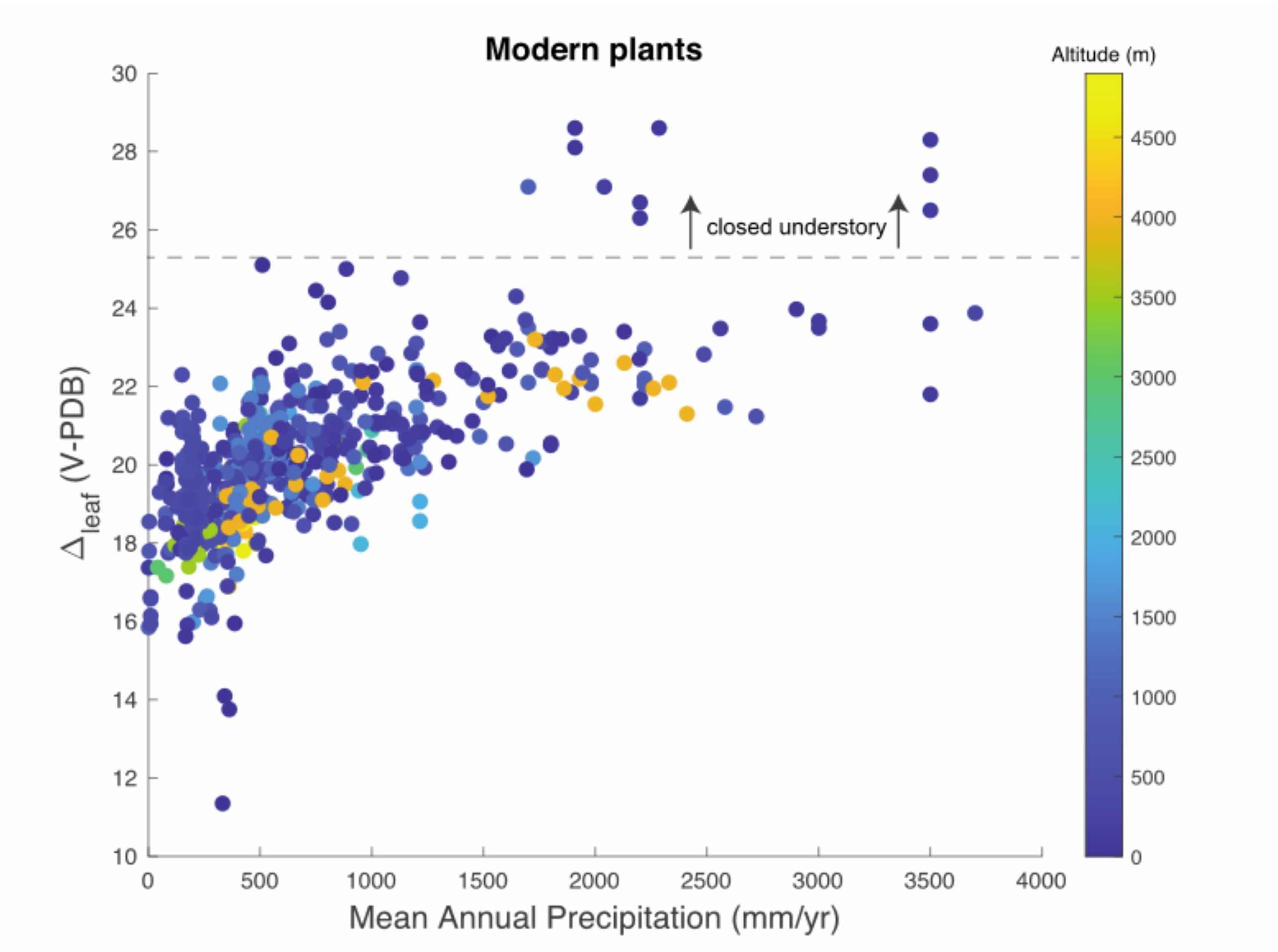

Supplementary figure 12. Carbon isotope fractionation, $\Delta_{leaf} = \delta^{13}C_{air} - \delta^{13}C_{leaf}$, in modern C3 plants versus mean annual precipitation, calculated using atmospheric $\delta^{13}C_{air}$ = -6.9‰ and $\delta^{13}C_{plant}$ data from refs. 35 and 36. Modern C3 plants in closed understory settings induce larger fractionations, but this effect is negligible for the low vegetation of the Early Devonian.

S2.8 Carbon isotope composition of the Early Devonian atmosphere

The carbon isotope composition of the atmosphere, $\delta^{13}C_{air}$, is in equilibrium with the oceanic pool of dissolved inorganic carbon pool that, in turn, is to first order determining the isotope composition of marine carbonates, $\delta^{13}C_{CARB}$. This is depicted in Supplementary fig. 13.

Diagenetic alteration can induce large overprints on $\delta^{13}C_{CARB}$ records, especially if altered by meteoric fluids. Therefore, it is important to compare data from several localities (Supplementary table 5). A temperature-dependent isotope fractionation occurs between aqueous CO_2 and marine carbonate precipitates. Our best estimate of $\delta^{13}C_{air}$ in Lower Devonian air is -6.4±0.5‰ through the 410-380 Ma interval shown in Supplementary fig. 9.

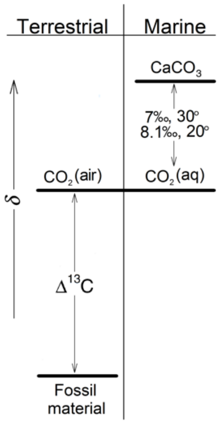

Supplementary figure 13. Carbon isotope fractionation between carbon in (fossil) terrestrial plants, CO_2 in air and marine carbonates (modified after ref. [37]).

	Locality	Age (Ma)	$\delta^{13}C_{CARB}$ † [‰]	$T_{avg.surf}$ ¥ (°C)	$\Delta^{13}C_{DIC-CO2(air)}$ *f* [‰]	$\delta^{13}C_{air}$ Ω [‰]	Refs
CE	Central Europe	Pragian	2.0±1.0	22.6±5.0	9.04±0.57	-5.8±1.2	A
NE	Nevada. USA	L. Pragian	1.0±1.0	21.8±5.0	9.13±0.57	-6.9±1.2	B
SI	Nizhnig Sergi and Tabulska. Siberia	Pragian	1.0±1.0	22.6±5.0	9.04±0.57	-6.8±1.2	C
OK	Haragan Bois d'Arc limestone. Oklahoma	Lochkovian	1.0±0.7	21.7±5.0	9.14±0.57	-6.1±0.9	D
	**Average**		**1.5±0.5**			**-6.4±0.5**	

Supplementary table 5. Carbon isotope composition of Early Devonian atmosphere derived from marine carbonates and the temperature-dependent carbon isotope difference between dissolved inorganic carbon (DIC) in seawater and atmospheric CO2. The data is plotted in the histogram of fig 1a.

† The range of carbon isotope values in marine limestones from the given time interval and location.
¥ Surface temperature estimates obtained from the Paleoclimate model with 500 ppm CO_2 (fig. 4) adopting a conservative uncertainty estimate of ±5°C.
f Equilibrium fractionation between dissolved inorganic carbon (DIC) and atmospheric CO_2 at the given range of temperatures according to Hayes 1999. ΩEstimated carbon isotope composition of atmospheric CO_2 using equation 10 in Hayes[37] assuming a constant isotopic offset between the studied limestone formations and open-ocean seawater of 1.2‰. The uncertainty estimate is propagated from the estimated temperature range and uncertainty in $\delta^{13}C_{CARB}$.
A – ref. (*29*), B – ref. (*30*), C – ref. *31*. D – ref.[41] (reporting an independent temperature estimate of T = 25±7°C from the Haragan Bois d'Arc limestone).

Section S3. Error propagation for other atmospheric pCO_2 proxies

S3.1 Stomatal proxies for paleoatmospheric CO_2

The density of stomata in land plants has been used to infer atmospheric CO_2 pressure in the Early Devonian[3]. A low stomatal density and a low stomatal index (SI = ratio of stomata to epidermis cells) in two extinct plants has been used to suggest higher atmospheric pCO_2 in the Early Devonian[32]. Both plant taxa were assumed to occupy poorly drained habitats in which the plants with minimal number of stomatal cells would have a selective advantage by preventing water loss[3]. However, lycophyte fossils of same age (Supplementary table 5) display similar stomatal density and size to modern lycophytes (Supplementary table 2) suggesting atmospheric pCO_2 levels were, in fact, similar to today.

We infer that the zosterophyll *Sawdonia ornata* and the non-vascular polysporangiophyte *Aglaophyton major* have no modern descendants that adequately can be used for the model calibration. To interpret ambient CO_2 levels from stomatal indices, the fossils data was compared to extant plants thought to represent their nearest living representative[32]. However, the stomatal density in Early Devonian *Baragwanathia* flora from Canada (SD = 28±5 mm^{-2}) and *Drepanophycus* (SD = 18±4 mm^{-2}) is higher than reported for these extinct plant taxa. A summary of stomata density data from Early Devonian plant fossils is given in Supplementary table 6 and can be compared to modern lycophytes (Supplementary table 2)[4]. Hence, low stomata density in extinct plant lineages is not sufficiently strong evidence for high atmospheric pCO_2 in the Lower Devonian.

Species	Age [Ma]	Stomatal density [mm^{-2}]	Ref.
**†Aglaophyton:**			
Aglaophyton major	Pragian-Emsian 410-393 Ma	4.5±3.9	Ref. [32]
**† Zosterophyllophyta:**			
Sawdonia ornata	Pragian-Emsian 410-393 Ma	4.3±1.9	Ref. [32]
**Lycopodiophyta:**			
Baragwanathia abitibiensis	Mid-Upp. Emsian ~400-393 Ma	28 ± 5	Ref. [8]
Drepanophycus spinnaeformis	Emsian ~405-401 Ma	16.6±2.0	Ref. [10]
Drepanophycus spinnaeformis	Givetian ~388 Ma	17.0±2.0	Ref. [10]
Drepanophycus spinnaeformis	Frasnian ~380 Ma	20.8±2.6	Ref. [10]

Supplementary table 6. Stomatal data from Early Devonian plants. Data from modern lycophytes is listed in Supplementary table 2.

We compared the stomatal density of the two living species of lycophytes, *Huperzia squarrosa* and *Huperzia phlegmaria*, grown at near-modern atmospheric pCO_2 (~448 ppm). Supplementary table 2 shows the stomatal densities in the modern lycophytes range from 20.1±2.8 to 28.7±2.3, indistinguishable or slightly higher than that observed in *Drepanophycus spinaeformis* and

Baragwanathia abitiensis. Therefore, the stomatal data is fully consistent with the low atmospheric pCO_2 estimate for the early Devonian atmosphere.

S3.2 Pedogenic goethite as proxy for palaeoatmospheric pCO_2

Ambient CO_2 concentrations influence the fraction, X, of CO_2 that can be substituted into goethite minerals during precipitation in soils; i.e. $X = FeCO_3OH / (FeOOH + FeCO_3OH)$. This phenomenon has been used to derive high atmospheric CO_2 concentrations from pedogenic goethite in a Late Ordovician paleosol from the Neda Fm in Wisconsin, USA[5]. The original paper reported a concentration of 4800 ppm and a recent error propagation calculation yields ±1900 ppm (1SD) [42]. This important constraint used in Earth System models depends on several assumptions and we show the error propagation of this calculation here.

The derivation of Late Ordovician atmospheric pCO_2 from pedogenic goethite relies on six major assumptions:

- The $\delta^{13}C$ of Late Ordovician atmosphere CO_2 is assumed to have been -6.4±1.1‰[5].

- Goethite precipitation incorporates CO_2 with an equilibrium isotopic fractionation that induce a constant 5.0‰ offset (no uncertainty) between the CO_2 in the mineral and ambient air.

- There is a positive trend of $\delta^{13}C$ vs. 1/X in pedogenic goethite from a paleosol profile at Potters Mill, which is assumed to represent a mixing line with atmospheric CO_2 and soil-respired CO_2 as the two end-member C sources, according to the following equation:

equation 4 $$\delta^{13}C = (\delta^{13}C_A - \delta^{13}C_O) \cdot X_A \cdot [1/X] + \delta^{13}C_O.$$

Data from the Potters Mill paleosol yields a slope and intercept ($\delta^{13}C = A \cdot /1/X) + B$) with the following uncertainties (2SE): $A = (\delta^{13}C_A - \delta^{13}C_O) \cdot X_A = 0.01594 \pm 0.00136$ and $B = \delta^{13}C_O = -20.39 \pm 0.35$

- Pedogenic goethites incorporate CO_2 in response to ambient CO_2 levels, according to the following equation:

equation 5 $$\log(X) = \log pCO_2 + \Delta S^0/(2.303 \cdot R) - \Delta H^0/(2.303 \cdot R)$$

where R is the universal gas constant and $\Delta S^0/(2.303 \cdot R) = 6.04$ (Yapp & Poths 1992)[43].

- The pedogenic goethite from Potters Mill are assumed to follow equation 5 for phosphatized goethite, $\Delta H^0 = 30$ kJ (and not for unphosphatized goethite, where $\Delta H^0 = 8$–16 kJ)[6]. The calculation is highly sensitive to this assumption (Supplementary fig. 14).

- Soil pH is assumed not to alter equation 5, even though it affects the proportion of CO_2 among dissolved inorganic carbon phases.

With these assumptions, the Potters Mills paleosol leads to an atmospheric pCO_2 constraint for the Late Ordovician of 4717^{+2066}_{-1270} ppm (3447–6784 ppm, 2 SD, hence 13–21 PIAL, 1 SD), excluding deviations from assumptions listed above. The is illustrated in Supplementary fig. 14.

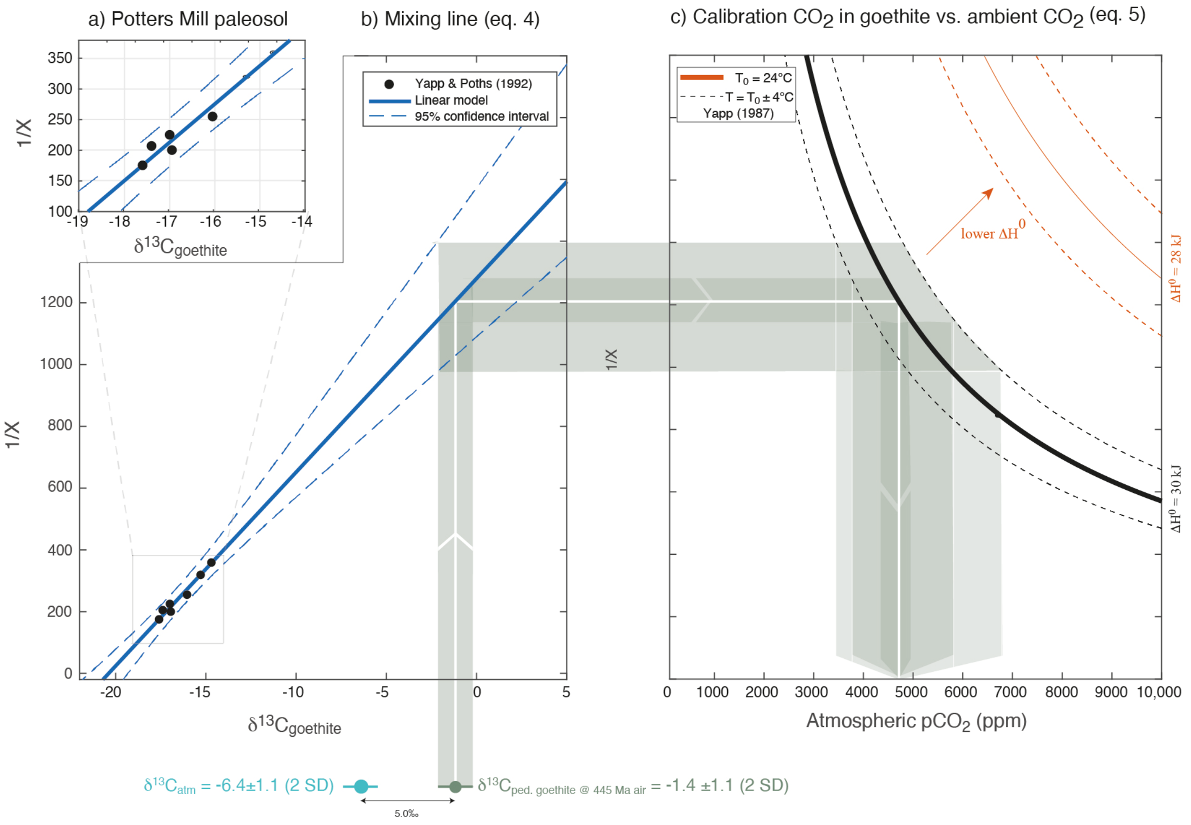

Supplementary figure 14. Illustrated error propagation of atmospheric pCO_2 constraint based on CO_2 incorporated into pedogenic goethite. A) Carbon isotope data ($\delta^{13}C$) and mole fraction of CO_2 in goethite (X = n(Fe(CO3)OH)/[n(FeOOH)+ n(Fe(CO_3)OH]) is inversely correlated in the Late Ordovician Potters mill paleosol. B) A linear fit is interpreted as a mixing line (eq. S1) that describes the isotopic consequences as a function of the relative proportion of CO_2 derived from soil respiration and from the atmosphere. Assuming the isotope composition of the Late Ordovician atmosphere ($\delta^{13}C_{atm}$) was -6.4‰, it is possible to derive the CO_2 mole fraction (X) that goethite would adopt if only exposed to atmospheric CO_2 (in the absence of soil respired CO_2). C) A thermodynamic relation exists between the molar content of CO_2 substituted into goethite and ambient CO_2 levels (eq. S2). This function is very sensitive to whether goethite is unphosphatized (lower ΔH_0).

S3.3 Paleosol carbonate proxy for paleoatmospheric CO_2

A soil-based CO_2 paleobarometer[44] has subsequently been applied to paleosols of the past 400 Ma[45]. Conceptually, the proxy is based on isotopic analyses of calcite that precipitates in soils while the soil is in direct communication with the atmosphere. The atmospheric CO_2 level is obtained by assuming that pedogenic calcite contains CO_2 derived from a binary mixing soil-respired CO_2 and atmospheric CO_2. The inferred atmospheric pCO_2 depends critically on how much CO_2 is contributed via soil respiration (Sz) and the isotope composition of soil-respired

CO_2. The value for Sz is kept fixed and typical values ranges from 440 to 50,000 ppm. Traditionally, Sz has been estimated by characterizing the soil type of the paleosol, but even if this is could be inferred for early Devonian paleosols, variations in soil respiration, depth of carbonate formation, and the effect of water stress affects the isotope composition of soil carbonate. There is large spatial $\delta^{13}C$ variability (~8‰) in soils from even the same region (e.g. ref. *23*), which likely relates to the various values of Sz. Here, we adopt a conservative error estimate of Sz only and shows that the pedogenic pCO_2 estimates for the Devonian carry large uncertainties, and a consistent with the new from fossil land plants.

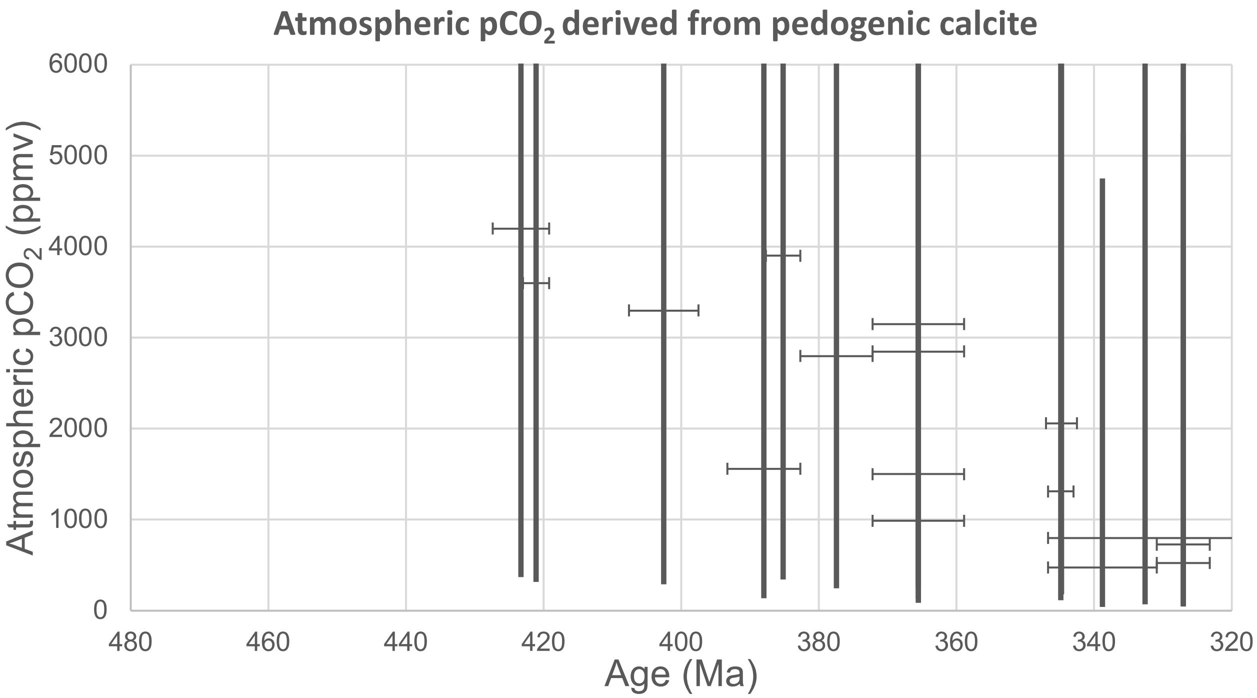

Supplementary figure 15. The carbon isotope composition of pedogenic calcite in paleosols also constrain atmospheric pCO_2. Errorbars represent the plausible range of CO_2 contributed from soil respiration (Sz), where Sz = 440-50,000 ppm with mid-point set to Sz = 2000 ppm as in Foster et al. 2017[26].

Section S4. Early Devonian Climate model.

S4.1 Early Devonian climate state

We simulated the Early Devonian climate using CLIMBER-3α (see Methods) to assess the climatic conditions at various CO_2 levels, and specifically the annual mean precipitation that influence the carbon isotope fractionation in land plants (Supplementary fig. 16).

First, three scenarios were explored where atmospheric CO_2 levels were fixed at 2000 ppm, 800 ppm and 500 ppm. In all scenarios, surface temperatures above 25°C occur in the tropics throughout the year and temperatures below freezing point occur in polar regions during winter. The global mean temperature declines from 22.1°C, 17.9°C to 15.0°C. Accordingly, the sea-ice fraction increases in the polar regions, respectively.

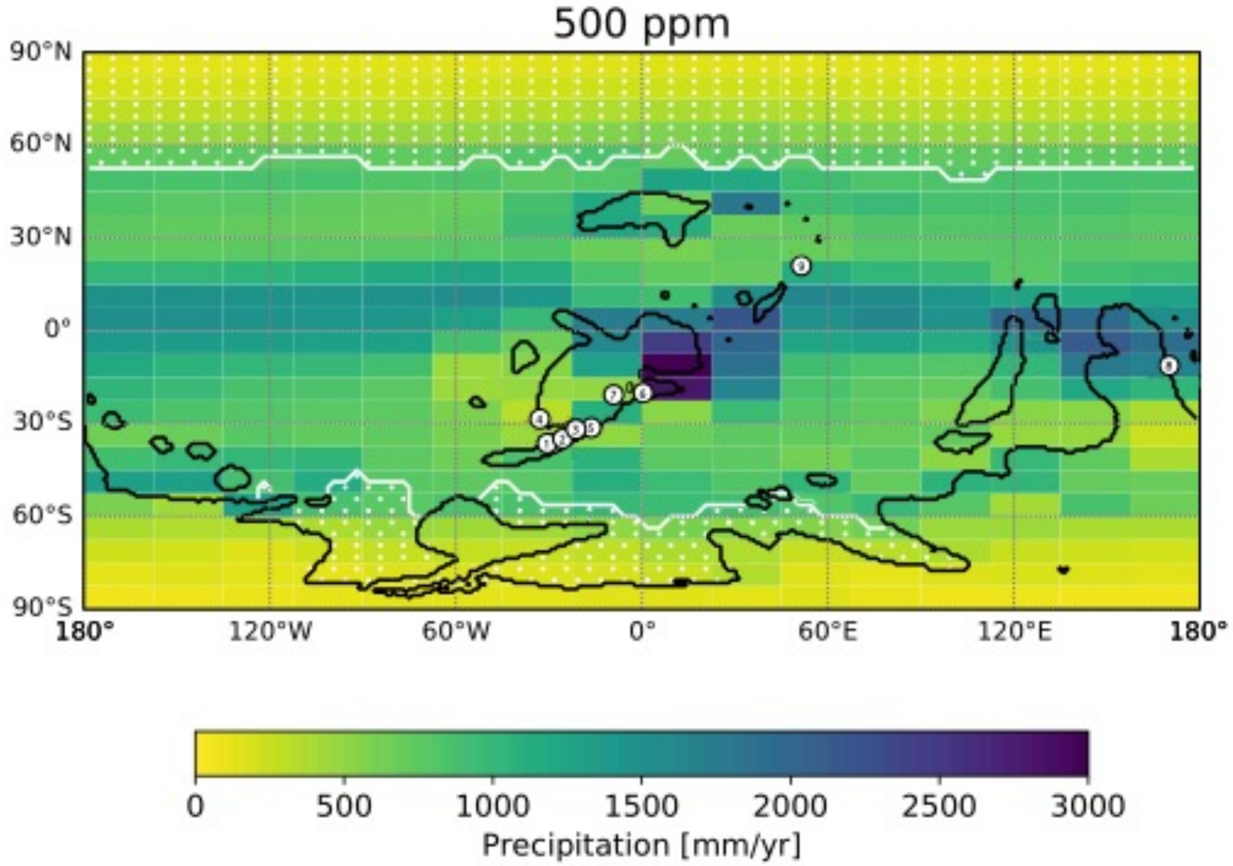

Supplementary figure 16. Mean annual rainfall (MAR) for the early Devonian (415 Ma) at atmospheric pCO_2 of 500 ppmv for the median orbit (obliquity of 23.5° and eccentricity of 0). Climate conditions in the fossil localities are listed in Supplementary Data 2. Mean annual temperatures are shown in fig. 3. Lycophyte fossil locations: 1 – Green County, NY, USA and Schoharia Co, NY, USA; 2 – Traveler Mountain, ME, USA; 3 – North Shore and Seal Rock, Gaspe Qbc., Canada; 4 – Abitibi River, Ontario, Canada; 5 – Dalhousie, New Brunswick, Canada (close to Maple Green); 6 – Munchshecke, Siegburg and Mosel Valley, Germany; 7 – Rhynie Chert, Aberdeenshire, Scotland; 8– Yea district, Victoria, Australia; 9 – Xinjan, China.

Subsequently, in line with our pCO_2 data we focused on the 500-ppm scenario and investigated the temperature distribution on the planet in detail for three different orbital configurations (Supplementary fig. 17). Sea ice accumulates in the polar ocean around Gondwana during Southern winters, whereas temperatures up to ~20°C occur in the continental interior during warmer summers (high obliquity and high eccentricity). The model considers snow cover on the continent but does not explicitly model ice sheet growth. Supplementary figure 18 shows duration of snow cover averaged over 1000 years for the three different orbital configurations. The permanence of polar ice sheets is not determined further here. However, the palaeoclimate conditions allow the presence of flora on Gondwana – even seasonally at high latitudes.

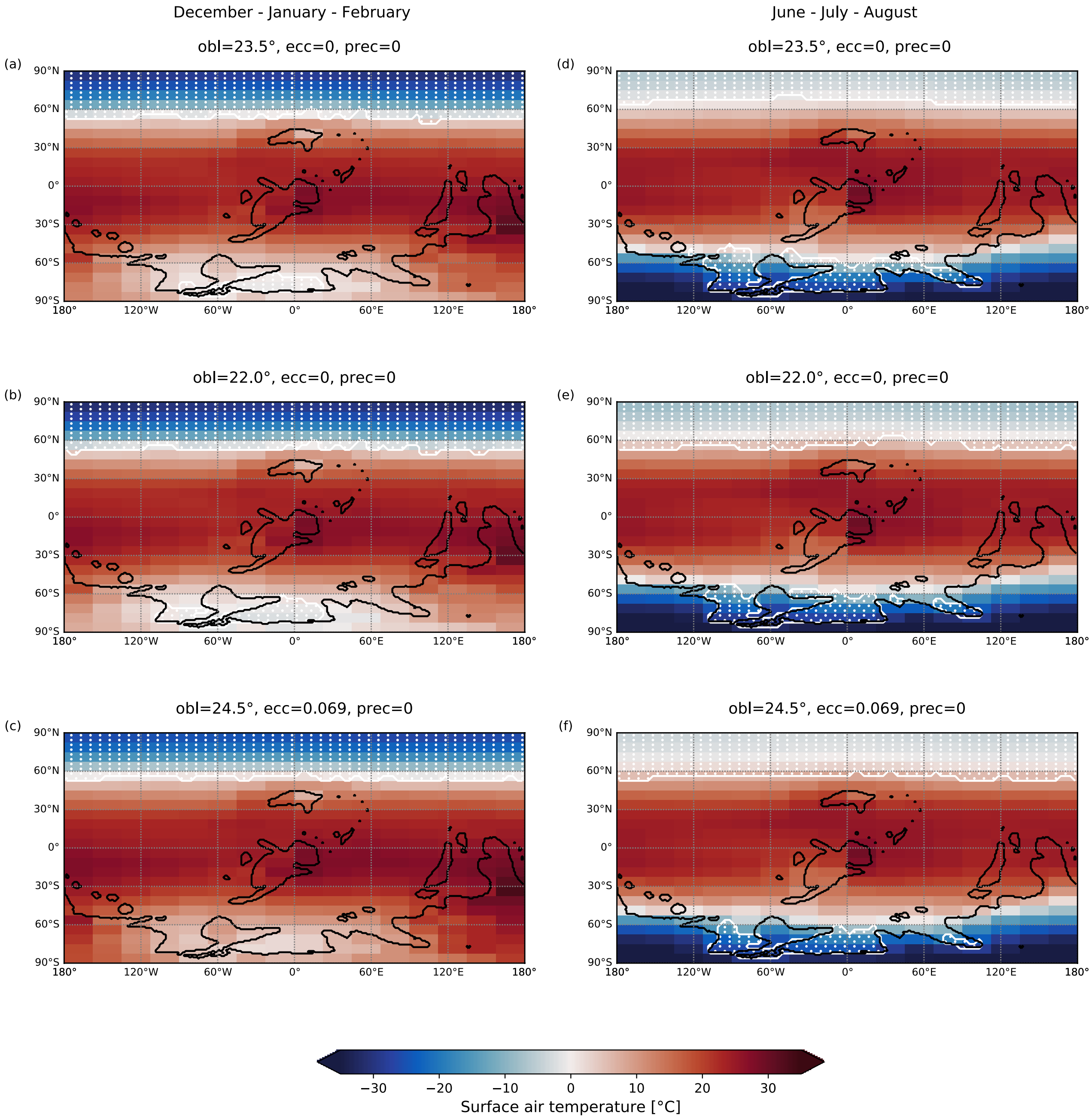

Supplementary figure 17. Devonian Palaeoclimate at 500 ppm atmospheric CO_2 for a-c) Southern summer and d-f) Southern winter. Three distinct orbital configurations are shown with a) and d) obliquity 23.5°, eccentricity 0; b) and e) obliquity 22.0°, eccentricity 0, and c) and f) obliquity 24.5°, eccentricity 0.069. The precession is 0 in all panels. NB! Polar sea ice occurs in all configurations.

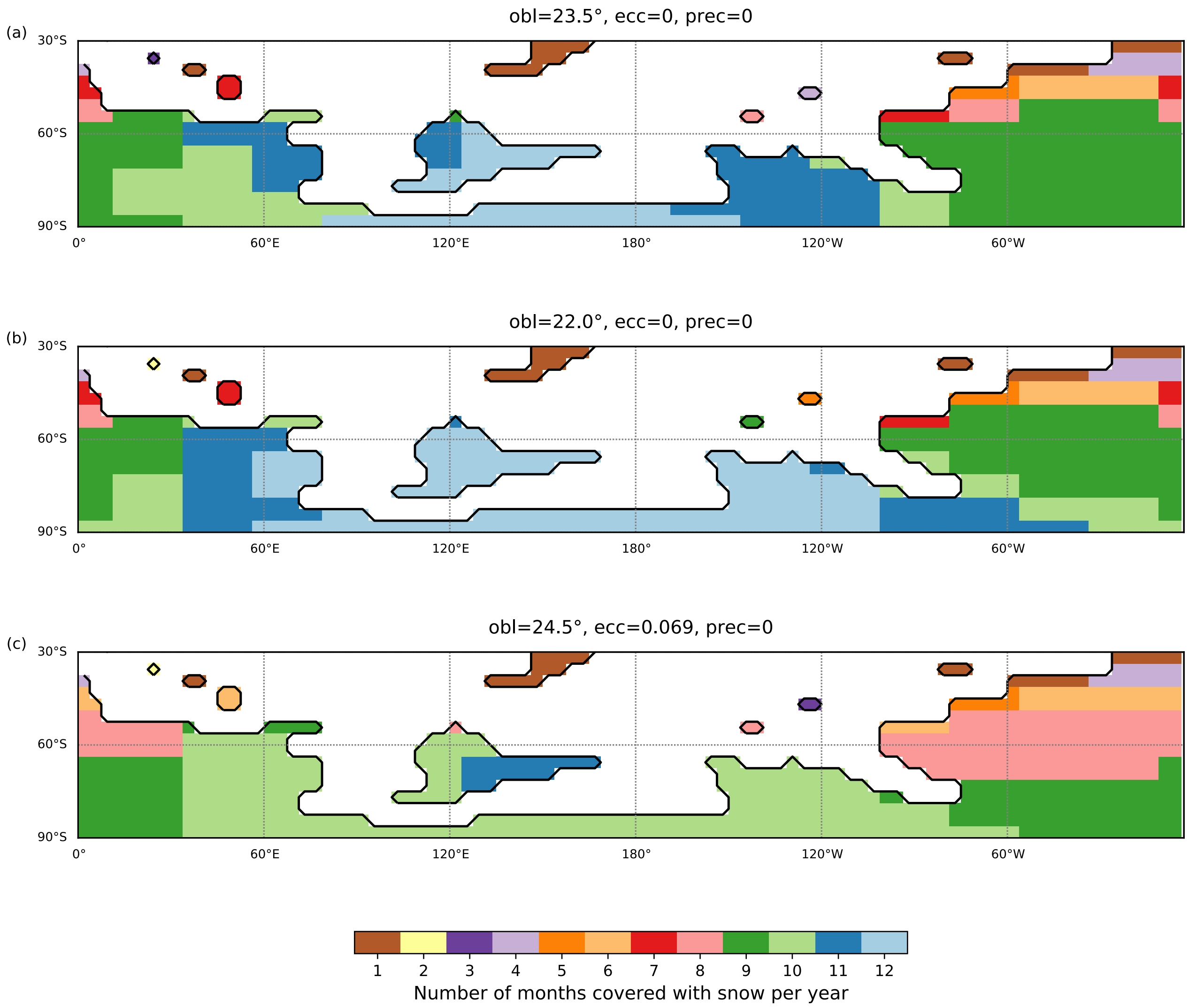

Supplementary figure 18. Duration of snow cover in Early Devonian Climate simulations with 500 ppm CO_2. Three distinct orbital configurations are shown with a) obliquity 23.5°, eccentricity 0; b) obliquity 22.0°, eccentricity 0, and c) obliquity 24.5°, eccentricity 0.069. The precession is 0 in all panels. The snow cover represents the average over 1000 years at the same three solar insolation configurations as in fig. Supplementary figure 17. The south pole is not permanently snow covered when obliquity = 24.5°.

S4.2. Sea Surface Temperatures in the Early and Middle Devonian

Our palaeoclimate model predicts Sea Surface Temperatures (SST) in the Devonian oceans that can be compared to proxy data obtained from the oxygen isotope composition of marine brachiopods and conodonts provided we know the $\delta^{18}O$ composition of the Devonian oceans in which the shells formed. Our focus here is to test how well the paleoclimate models with 500 ppm and 2000 ppm CO_2 fit the proxy data. The tabulated data and comparison of SSTs is summarized in Supplementary Data 2.

We compiled $\delta^{18}O_{calcite}$ data from well-preserved (e.g. non-luminescent shells) brachiopods and computed the average, minimum and maximum paleo-SSTs from each location. The proxy predicts SST from the T-$d^{18}O_{calcite}$ relationship ($T_{calcite,ice-free} = 3.17 + 4.95 \cdot(\delta^{18}O_{seawater} - \delta^{18}O_{calcite})$[47] where seawater is traditionally assumed to have a composition of $d^{18}O_{seawater}$ = -1.1‰ under modern-day, albeit ice-free, conditions. We will test this assumption below, since the $\delta^{18}O$ composition of seawater has increased over the course of Earth history and Devonian seawater likely had significant lower $\delta^{18}O_{seawater}$ value (-4 to -2‰) than assumed (-1.1‰) today[48], corresponding to substantial overestimates (~4-12°C) of the proxy temperatures below.

Assuming $\delta^{18}O_{seawater}$ = -1.1‰, paleo-SSTs from tropical Europe (Eifelian Mountains, Germany, $T_{calcite,ice-free}$ = 23±1°C, 1 SD, n =15) fit with average paleo-SST temperatures obtained from our paleoclimate model with an atmospheric pCO_2 levels of 500 ppm (20±3°C) as well as scenarios with 2000 ppm (24±2°C). Here, the model SST ranges represent the annual variation. Very high paleotemperatures are reported from brachiopods found in two subtropical locations from Iowa, USA ($T_{calcite,ice-free}$ = 35±3°C, 1 SD, n =20 in Buffalo Quarry and $T_{calcite,ice-free}$ = 30±2°C, 1 SD, n =2; Glorry Quarry) where our paleoclimate model predicts mean SSTs of 16-22°C and 20-26 °C for the 500 ppm and 2000 ppm CO_2, respectively. Hence, none of the models fit the proxy data even when accounting for $\delta^{18}O$ change on a glaciated Earth. Perhaps, we have assigned incorrect paleo-latitude or the shells have been chemically alterated. Further, our climate model predicts significantly lower SST in the temperate zone of the Cantabrian Mountains, Spain (1-13°C) and Anti-Atlas Mountains, Morocco (-1 to 8°C) at 500 ppm compared to the model at 2000 ppm CO_2 (at ~10-19°C and 5-15°C, respectively), but the proxy estimates of well-preserved brachiopods span a wide range of values (-6°C to +30°C and -13°C to 30°C; respectively) consistent with both scenarios, also if we use the $SST_{calcite}$ equation for Earth in a (modern-like) glaciated climate state. In summary, our model shows that the biggest difference in sea surface temperatures occurs at higher latitudes, but the current paleotemperature records are too sparse to verify this.

Further, we computed the average, minimum and maximum paleo-SSTs from each location with more than one data point from phosphatic conodonts using the T- $\delta^{18}O_{phos}$ relationship, $T_{phos} = a - b \cdot(d^{18}O_{phos} - d^{18}O_{seawater})$[49], assuming Devonian had a fixed seawater composition $\delta^{18}O_{SW}$. The uncertainties (1SD) of SSTs were calculated by error propagation of the uncertainty of the parameter values (a =118.7±4.9°C, b = 4.2±0.2; $\delta^{18}O_{SW}$ of -1.1±0.0‰ VSMOW). Typical SST absolute errors are relatively large, ±6.3°C (1SD), and therefore most of the predicted SSTs from fossils are also compatible with the paleoclimate model with either 500 ppm or 2000 ppm atmospheric CO_2.

In tropical Gondwana, the average T_{phos} from Pragian (33±6°C, n=39) and Emsian (29±6°C, n=39) deposits in Victoria, Australia as well as Lochkovian (29±14°C, n=18), Emsian (30±6°C, n=5) and Eifelian deposits (29±6°C, n=4) in Queensland, Australia are all compatible with the 500 ppm model scenario (27±3°C) as well as the 2000 ppm scenario (30±2°C). In tropical South China (Guangxi and Yunnan Province), the recorded T_{phos} from Emsian deposits (27±6°C, n =20 at Changputang; 27±6°, n= 3 at Nayi, and 25±6°C, n=13 at Sihongshan), and Givetian deposits (34±6°C, n = 8 at CaiZiyan, and 26±6°C at Changputang)[47,50] fit or nearly fit modelled SSTs ranging from 24 to 27 °C with the 410 Ma paleocontinental configuration. Eifelian deposits at Sihongshan yield lower proxy temperatures (20±6°C, n =14) only compatible with the 500 ppm

model (22-27°C) and incompatible with predicted SST for the model with 2000 ppm CO_2 (27-29°C). Contrary, at CaiZiyan in the Guangxi Province, Givetian conodonts suggest higher sea-surface temperatures of 30-42°C than predicted with either a 2000 ppm CO_2 paleoclimate model (21-26°C) or 500 ppm CO_2 paleoclimate model (19-22°C). In tropical western Laurentia (Nevada, USA), Emsian-Eifelian proxy-SST data (29±6°C)[51] is compatible with both model scenarios. We did not explore whether the modelled SSTs would change dramatically under different paleogeography, but we consider our conclusions robust at the given level of precision.

In Europe, conodonts from the Prague basin, Czech Republic, indicate relatively warm waters in the Lochkovian (30±6°C, n=37) and somewhat lower temperatures in the Emsian (22±6°C, n =28) and Eifelian (23±14°C, n=16). This locality was in the temperate zone (~45°S, GPlates) and the model predicts SSTs at 13±4 °C and 19+4°C for the 500 ppm and 2000 ppm scenarios, respectively[51]. Similarly, conodonts from the temperate climate zone in France (e.g. Puech de la Suque) yields SSTs for the Emsian (22±6°C, n= 2), Eifelian (21±6°C, n= 8), and Givetian (21±6°C, n= 38) that are distinctly warmer than the 500 ppm CO_2 model scenario (1-13°C) and compatible with the 2000 ppm scenario (10-19°C) considering the large uncertainty of the temperature equation.

In the subtropical zone of Laurentia, conodont samples from Iowa suggest Givetian SST was 29±6°C, n=22 (compatible with the high temperatures inferred from well-preserved brachiopods in the same area), and consistent with the modelled SST of 19±4°C and 23±3°C for the 500 ppm and 2000 ppm scenario, respectively.

In summary, assuming of Devonian seawater had a composition of $\delta^{18}O_{seawater}$ = -1.1‰, our paleoclimate model with 500 ppm fits with proxy SST data in 18 out of 30 deposits, whereas the 2000 ppm CO_2 scenario fits a few more sites (22) mainly located at higher latitudes. We find that the cumulative misfit (sum of offsets at all 30 deposits) would be minimal with a Devonian seawater $\delta^{18}O_{seawater}$ of -2.9‰ and -1.9‰ for the 500 ppm and 2000 ppm scenarios, respectively. Assuming the optimal $\delta^{18}O_{seawater}$ values for Devonian seawater, then the predicted sea-surface temperature would fit at almost all sites and approximately equally well; i.e. 26 and 27 sites out of 30 for the 500 and 2000 ppm scenarios, respectively (Supplementary Data 2). According to our model scenarios, we conclude that more (precise) SST constraints (incl. $\delta^{18}O_{seawater}$) – and preferably from localities in the temperate climate zones would best constrain past CO_2 level. At present, we conclude that both the 500 ppm and the 2000 ppm CO_2 model scenarios fit the existing paleo sea-surface temperature estimates equally well.

S4.3 Mean Annual Precipitation at the Devonian fossil localities

The paleoclimate model simulations provide mean annual rainfall (MAR) for the Yea fossil site in Victoria, Australia, and other localities where fossil lycophytes have been found (Supplementary Data 2). For all realistic atmospheric pCO_2 levels >500 ppm, the flora would have grown in humid tropical settings with ~2 times higher MAR than the threshold of ~1000 mm/yr where modern C3 plants begin to upregulate WUE with concomitant smaller C isotope fractionation. Other Devonian lycophyte fossil sites occur in the drier subtropics and show smaller average MAR values at the interpolated grid of 1x1° resolution. Nevertheless, precipitation and humidity can vary on smaller geographical scales, and hence all lycophyte

floras may have been living in sufficient wet (optimal) conditions. Therefore, the Devonian lycophytes should reliably record atmospheric CO_2, as also indicated by having similar stomatal anatomy to modern lycophytes.

Obliquity (ε), eccentricity (e), precession (ϖ)	pCO_2 [ppm]	T_s(°C)	MAR (mm/yr)	RH
ε = 23.5°, e = 0, ϖ = 0	500	27.1	1640	0.65
ε = 22.0°, e = 0, ϖ = 0	500	27.2	1699	0.65
ε = 24.5°, e = 0.069, ϖ = 0	500	27.5	1658	0.65
ε = 23.5°, e = 0, ϖ = 0	800	29.4	1909	0.62
ε = 23.5°, e = 0, ϖ = 0	2000	33.0	2372	0.59

Supplementary table 7. Mean annual surface-air temperature T_s, mean annual rainfall (MAR) and relative humidity (RH) for the Baragwanathia flora at the Yea fossil site in Victoria, Australia, according to the paleoclimatic model (CLIMBER-3α), interpolated to a 1-by-1 degree grid.

Section S5. Revised COPSE model for the Mid-Paleozoic

To evaluate conditions that might have facilitated low atmospheric CO_2 in the Devonian, we used the Carbon-Oxygen-Phosphorous-Sulfur Evolution (COPSE) Earth system model framework with adjusted forcing parameters on weathering to fit simultaneously fit observational constraints on atmospheric pCO_2 and pO_2. We adapted the latest version of the COPSE model, COPSEreloaded (CR), which has slightly different parameterizations of climate sensitivity, continental weathering, and ocean anoxia compared to previous COPSE models[52,53]. The revised CR model is denoted 'CR2022' and is plotted in Supplementary figs. 19–21 to show the modifications made to fit the new observational constraints.

The new observational constraints demand lower atmospheric pCO_2 levels in the Early Devonian than in any previous models. In CR, atmospheric CO_2 levels drops from ~3300 ppm to a plateau at 1900 ppm (12→7 PAL, 1 PAL is taken to be 280 ppm) between 465 and 445 million years ago (Ma), which is followed by a further decline beginning at 400 Ma and reaching 360 ppm at 360 Ma[53]. In C16, atmospheric CO_2 levels declines from ~4500 to ~2800 ppm between 460 Ma and 445 Ma, which is followed by a slower decline from 2800 ppm to 1680 ppm (~10→6 PAL) until 360 Ma[52]. The GEOCARB family of models predicts even higher atmospheric pCO_2 levels 410–380 Ma, incl. 2500-3000 ppm (GEOCARB I, 9-11 PAL[19]), ~3300 ppm (GEOCARB II, 12 PAL[54]), and ~2800-4200 ppm (10-15 PAL in GEOCARB III[11]/15/22 10:58:00 PM and GEOCARBSULF[23]), although the error range is large (800-8000 ppm)[27]. We revised the CR model to meet the new observational pCO_2 proxy constraints (440-640 ppm, 410-380 Ma) mainly by revisiting the parameterization of how vascular plants evolved and affected continental weathering processes and correction to the volcanic outgassing rate.

First of all, we let the effects of plant-enhanced silicate (and carbonate) weathering scale with mudrock proportion in continental deposits, normalized to the average Carboniferous value (mudrock% = 27). This is justified because mineral surface area and plant-assisted suspension of finer grains exposed to weathering fluids (baffling) would increase the rates of rock dissolution. The effect of coupling W linearly to mudrock proportion makes only a small difference in the pCO_2 curve relative to the original CR model, simply because the original W forcing is actually very similar (Supplementary fig. 19).

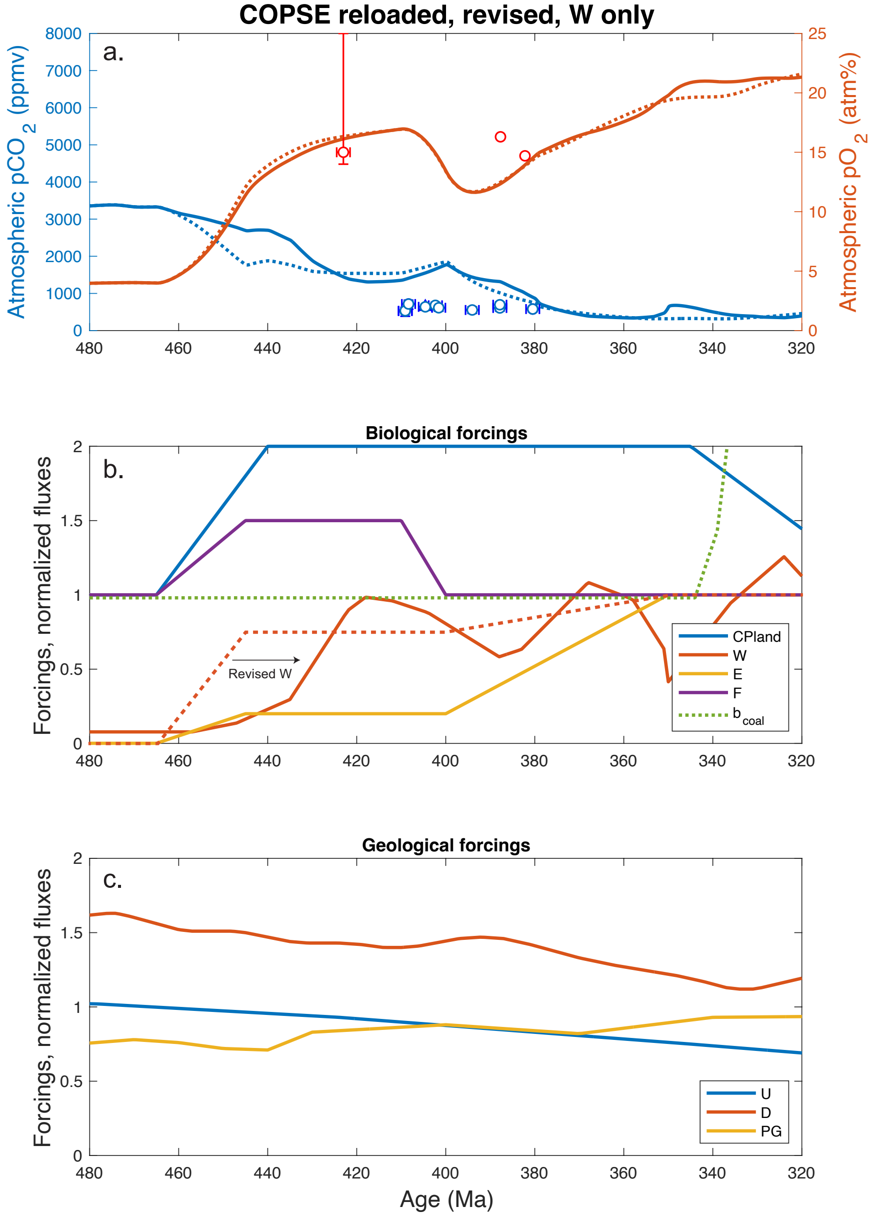

Supplementary figure 19. COPSE Reloaded model simulations for the evolution of the global biogeochemical cycles are shown, where a) shows the atmospheric pO_2 and pCO_2 trajectories, b) the biological forcings (CPland – C/P ratio of terrestrial biomass, W– weathering, E– evolution of terrestrial vegetation, F– selective P weathering and b_{coal} – coal deposition) and c) geological forcings (U –Uplift, D– volcanic degassing, and PG – paleogeography). The baseline CR model is shown (dotted curves) for comparison to the CR model with revised weathering forcing (W). The observational constraints on pO_2 and pCO_2 from the charcoal record[55,56] and the calibrated pCO_2 proxy data are shown in a.

Secondly, the evolution of land plants (forcing E) in the Silurian is ramped up earlier than in CR to mimic that plant-assisted weathering became more widespread earlier than previously assumed. Plant coverage is difficult to constrain directly by observation, but trait-based spatial modeling of cryptogamic vegetation (i.e., bryophyte and lichen) cover predicts the potential global net primary productivity (NPP) was 30% of today's level at 8 PAL (2240 ppm) in the Late Ordovician[57]. Further, lycophyte vegetation could be even more productive[58]. Presumably, NPP further increased as vascular plants evolved. Because plant evolution has important consequences for the magnitude of the pO_2 rise and pCO_2 drawdown in the COPSE model, we tuned up Silurian plant coverage to ~50% of today's coverage which dramatically affects the pO_2 rise and pCO_2 drawdown (Supplementary fig. 20).

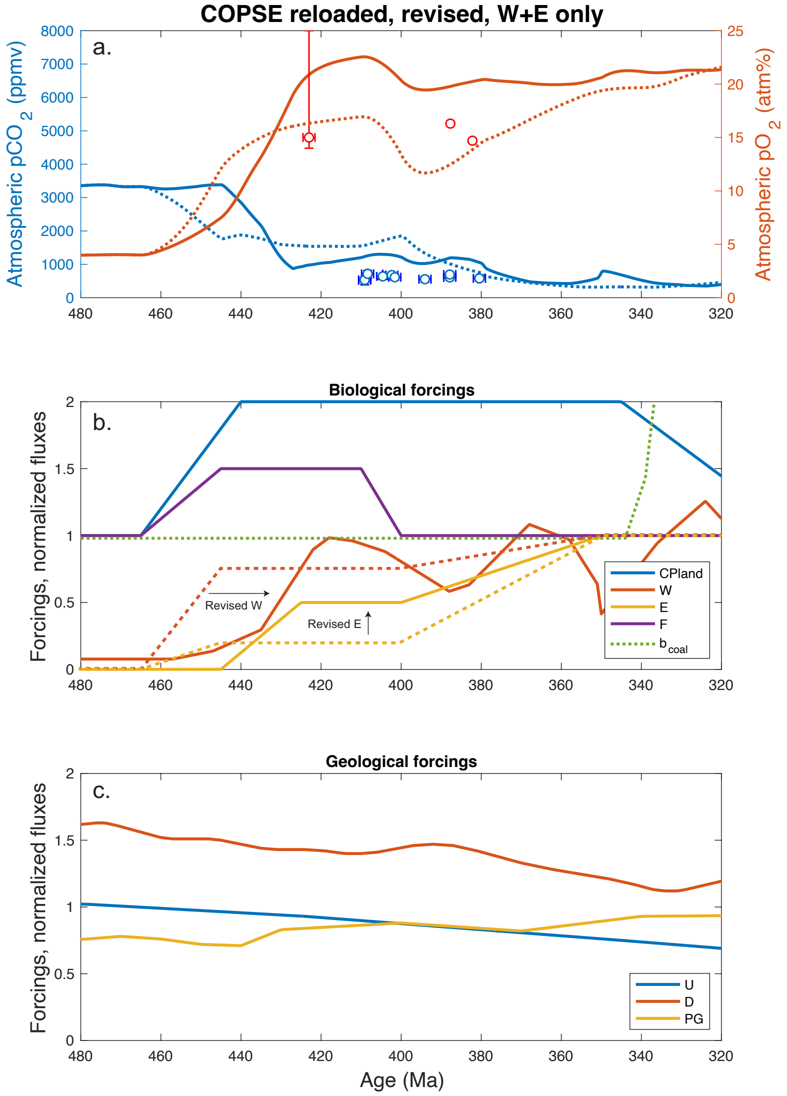

Supplementary figure 20. COPSE reloaded simulations (as in Supplementary fig. 19) with revised forcing of Weathering (W) and plant evolution (E)[53]. Panels show a) predicted atmospheric pO_2 and pCO_2 trajectories, b) the biological forcings and c) geological forcings as in Supplementary fig. 19. The E-forcing accounts for the (CO_2-and temperature-sensitive) biotic enhancement on weathering induced by terrestrial plants compared to plant-free environments (e.g. $k_{preplant}/k_{plant} = 0.15$).

Recent versions of the COPSE model[52,53,59] take selective P weathering (forcing F) relative to bulk cations (e.g., Ca, Si) into account for primitive land plants. Experiments with mycorrhizal liverworts demonstrate that non-vascular plants can selectively 'mine' P from a geological substrate. This may have been particularly pertinent when early plants were colonising fresh rock surfaces before the establishment of rooting systems, deeper soils and effective P recycling systems. In the revised CR model, we let selective P weathering of the early vascular flora evolve mirror that or bryophytes as in CR, which also means in parallel to the higher C/P ratio in terrestrial biomass.

Third, we adjusted the CO_2 degassing rate to scale with emissions from continental arc volcanism rather than seafloor spreading rates. This is justified because most CO_2 emanating from Earth's interior comes from subducted carbonate platforms and is not solely coupled to global seafloor spreading rates as was assumed in previous COPSE models[52,53,60,61]. The volcanic degassing (D) forcing is adjusted to scale linearly with Continental Volcanic Arc (CVA) emissions that, in turn, scales in proportion to "Young vs. Old" grains in sedimentary deposits [62]. With modern day set to 1, we find that degassing rates varied between 1.0 and 1.2 during 480–320 Ma and that the volcanic CO_2 emission flux was 20% lower than assumed in the COPSE Reloaded baseline model (Supplementary fig. 21).

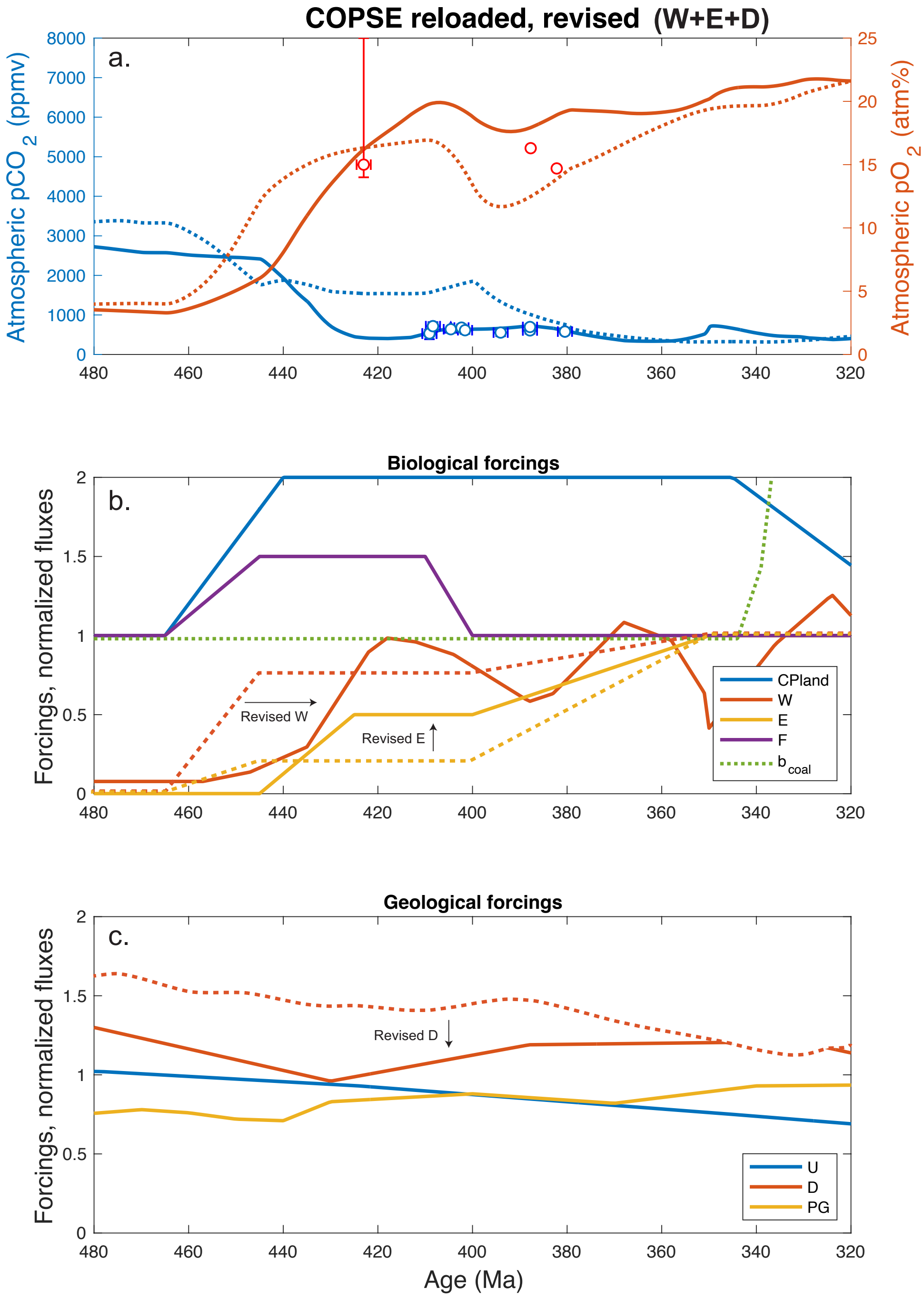

Supplementary figure 21. COPSE reloaded simulations (as in Supplementary fig. 19) with revised forcing of Weathering (W), Plant evolution (E), selective weathering (F), and volcanic outgassing (D) scaled linearly to Continental Volcanic Arc (CVA) emission, which in turn is scaled in proportion to Young vs. Old grains in sedimentary deposits[62]. Panels show a) predicted atmospheric pO_2 and pCO_2 trajectories, b) the biological forcings and c) geological forcings as in Supplementary figs. 19–20.

Supplementary references

1. Garratt, M. J. New evidence for a Silurian (Ludlow) age for the earliestBaragwanathiaflora. *Alcheringa: An Australasian Journal of Palaeontology* **2**, 217–224 (1978).
2. Tims, J. The early land flora of Victoria. (The University of Melbourne, 1980).
3. Rickards, R. B. The age of the earliest club mosses: the Silurian *Baragwanathia* flora in Victoria, Australia. *Geol. Mag.* **137**, 207–209 (2000).
4. Earp, C. *Costulatotheca schleigeri* (Hyolitha: Orthothecida) from the Walhalla Group (Early Devonian) at Mount Pleasant, central Victoria, Australia. *Alcheringa: An Australasian Journal of Palaeontology* **43**, 220–227 (2019).
5. Cookson, I. IV—On plant-remains from the Silurian of Victoria, Australia, that extend and connect floras hitherto described. *Phil. Trans. R. Soc. Lond. B* **225**, 127–148 (1935).
6. Kraft, P. & Kvaček, Z. Where the lycophytes come from? – A piece of the story from the Silurian of peri-Gondwana. *Gondwana Research* **45**, 180–190 (2017).
7. Franks, P. J. *et al.* New constraints on atmospheric CO_2 concentration for the Phanerozoic. *Geophys. Res. Lett.* **41**, 4685–4694 (2014).
8. Hueber, F. M. A new species of Baragwanathia from the Sextant Formation (Emsian) Northern Ontario, Canada. *Botanical Journal of the Linnean Society* **86**, 57–79 (1983).
9. Brodribb, T. J. & Holbrook, N. M. Declining hydraulic efficiency as transpiring leaves desiccate: two types of response. *Plant Cell Environ* **29**, 2205–2215 (2006).
10. Edwards, D. Stomata in early land plants: an anatomical and ecophysiological approach. *Journal of Experimental Botany* **49**, 255–278 (1998).
11. Boyce, C. K. *et al.* Chemical Evidence for Cell Wall Lignification and the Evolution of Tracheids in Early Devonian Plants. *International Journal of Plant Sciences* **164**, 691–702 (2003).
12. Wan, Z. *et al.* Environmental influences on the stable carbon isotopic composition of Devonian and Early Carboniferous land plants. *Palaeogeography, Palaeoclimatology, Palaeoecology* **531**, 109100 (2019).
13. Cramer, B. D. & Jarvis, I. Carbon Isotope Stratigraphy. in *Geologic Time Scale 2020* 309–343 (Elsevier, 2020). doi:10.1016/B978-0-12-824360-2.00011-5.
14. Stubblefield, S. & Banks, H. P. The Cuticle of Drepanophycus Spinaeformis, a Long-Ranging Devonian Lycopod from New York and Eastern Canada. *Am. J. Botany* **65**, 110–118 (1978).
15. Kennedy, K. L., Gensel, P. G. & Gibling, M. R. Paleoenvironmental inferences from the classic Lower Devonian plant-bearing locality of the Campbellton Formation, New Brunswick, Canada. *PALAIOS* **27**, 424–438 (2012).
16. Franks, P. J. & Royer, D. L. Comment on "Was atmospheric CO2 capped at 1000 ppm over the past 300 million years?" by McElwain J. C. et al. [Palaeogeogr. Palaeoclimatol. Palaeoecol. 441 (2016) 653–658]. *Palaeogeography, Palaeoclimatology, Palaeoecology* **472**, 256–259 (2017).
17. Cheng-Sen, L. A re-investigation of Halle's Drepanophycus spinaeformis Göpp. from the Lower Devonian of Yunnan Province, southern China. *Botanical Journal of the Linnean Society* **118**, 163–192 (1995).
18. Berner, R. A. Atmospheric Carbon Dioxide Levels Over Phanerozoic Time. *Science* **249**, 1382–1386 (1990).
19. Berner, R. A. A model for atmospheric CO2 over Phanerozoic time. *Am J Sci* **291**, 339–376 (1991).
20. Berner, R. A. Paleozoic Atmospheric CO2: Importance of Solar Radiation and Plant Evolution. *Science* **261**, 68–70 (1993).
21. Berner, R. A., Lenton, T., Tester, M. & Beerling, D. J. The Carbon Cycle and CO2 Over Phanerozoic Time: The Role of Land Plants. *Philosophical Transactions: Biological Sciences* **353**, 75–82 (1998).

22. Berner, R. A. & Kothavala, Z. GEOCARB III: A revised model of atmospheric CO2 over Phanerozoic time. *American Journal of Science* **301**, 182–204 (2001).
23. Berner, R. A. GEOCARBSULF: A combined model for Phanerozoic atmospheric O2 and CO2. *Geochimica et Cosmochimica Acta* **70**, 5653–5664 (2006).
24. Nardin, E. *et al.* Modeling the early Paleozoic long-term climatic trend. *Geological Society of America Bulletin* **123**, 1181–1192 (2011).
25. Berner, R. A. The Rise of Plants and Their Effect on Weathering and Atmospheric CO$_2$. *Science, New Series* **276**, 544–546 (1997).
26. Foster, G. L., Royer, D. L. & Lunt, D. J. Future climate forcing potentially without precedent in the last 420 million years. *Nat Commun* **8**, 14845 (2017).
27. Royer, D. L., Donnadieu, Y., Park, J., Kowalczyk, J. & Godderis, Y. Error analysis of CO2 and O2 estimates from the long-term geochemical model GEOCARBSULF. *American Journal of Science* **314**, 1259–1283 (2014).
28. Porter, A. S., Evans-Fitz.Gerald, C., Yiotis, C., Montañez, I. P. & McElwain, J. C. Testing the accuracy of new paleoatmospheric CO2 proxies based on plant stable carbon isotopic composition and stomatal traits in a range of simulated paleoatmospheric O2:CO2 ratios. *Geochimica et Cosmochimica Acta* **2019**, 69–90 (2019).
29. Boyce, C. K. *et al.* Devonian landscape heterogeneity recorded by a giant fungus. *Geol* **35**, 399 (2007).
30. Saltzman, M. R. & Thomas, E. Carbon Isotope Stratigraphy. in *The Geologic Time Scale* 207–232 (Elsevier, 2012). doi:10.1016/B978-0-444-59425-9.00011-1.
31. Sun, T.-X., Edwards, D. & Cheng-Sen, L. The stomatal apparatus of Lycopodium japonicum and its bearing on the stomata of the Devonian lycophyte Drepanophycus spinaeformis. *Botanical Journal of the Linnean Society* **149**, 209–216 (2005).
32. McElwain, J. & Chaloner, W. Stomatal Density and Index of Fossil Plants Track Atmospheric Carbon Dioxide in the Palaeozoic. *Annals of Botany* **76**, 389–395 (1995).
33. Franks, P. J. & Farquhar, G. D. The Mechanical Diversity of Stomata and Its Significance in Gas-Exchange Control. *Plant Physiology* **143**, 78–87 (2007).
34. Farquhar, G., O'Leary, M. & Berry, J. On the Relationship Between Carbon Isotope Discrimination and the Intercellular Carbon Dioxide Concentration in Leaves. *Functional Plant Biol.* **9**, 121 (1982).
35. Kohn, M. J. Carbon isotope compositions of terrestrial C3 plants as indicators of (paleo)ecology and (paleo)climate. *Proceedings of the National Academy of Sciences* **107**, 19691–19695 (2010).
36. Diefendorf, A. F., Mueller, K. E., Wing, S. L., Koch, P. L. & Freeman, K. H. Global patterns in leaf 13C discrimination and implications for studies of past and future climate. *Proceedings of the National Academy of Sciences* **107**, 5738–5743 (2010).
37. Hayes, J. M., Strauss, H. & Kaufman, A. J. The abundance of 13C in marine organic matter and isotopic fractionation in the global biogeochemical cycle of carbon during the past 800 Ma. *Chemical Geology* **161**, 103–125 (1999).
38. Buggisch, W. & Joachimski, M. M. Carbon isotope stratigraphy of the Devonian of Central and Southern Europe. *Palaeogeography, Palaeoclimatology, Palaeoecology* **240**, 68–88 (2006).
39. Saltzman, M. R. Carbon isotope (N13C) stratigraphy across the Silurian^Devonian transition in North America: evidence for a perturbation of the global carbon cycle. 18.
40. Veizer, J. *et al.* 87Srr86Sr, d13C and d18O evolution of Phanerozoic seawater. 30 (1999).
41. Gao, G. The temperatures and oxygen-isotope composition of early Devonian oceans. *Nature* **361**, 712–714 (1993).
42. Dahl, T. W. & Arens, S. K. M. The impacts of land plant evolution on Earth's climate and oxygenation state – An interdisciplinary review. *Chemical Geology* **547**, 119665 (2020).
43. Yapp, C. J. & Poths, H. Ancient atmospheric CO2 pressures inferred from natural goethites. *Nature* **355**, 342–344 (1992).
44. Cerling, T. E. Carbon dioxide in the atmosphere; evidence from Cenozoic and Mesozoic

Paleosols. *American Journal of Science* **291**, 377–400 (1991).
45. Ekart, D. D. A 400 million year carbon isotope record of pedogenic carbonate; implications for paleoatomospheric carbon dioxide. *American Journal of Science* **299**, 805–827 (1999).
46. Rugenstein, J. K. C. *et al.* The Neogene de-greening of Central Asia. *Geology* **44**, 887–890 (2016).
47. Grossman, E. L. & Joachimski, M. M. Oxygen Isotope Stratigraphy. in *Geologic Time Scale 2020* 279–307 (Elsevier, 2020). doi:10.1016/B978-0-12-824360-2.00010-3.
48. Galili, N. *et al.* The geologic history of seawater oxygen isotopes from marine iron oxides. *Science* **365**, 469–473 (2019).
49. Pucéat, E. *et al.* Revised phosphate–water fractionation equation reassessing paleotemperatures derived from biogenic apatite. *Earth and Planetary Science Letters* **298**, 135–142 (2010).
50. Chen, B. *et al.* Devonian paleoclimate and its drivers: A reassessment based on a new conodont δ18O record from South China. *Earth-Science Reviews* **222**, 103814 (2021).
51. Elrick, M. *et al.* Stratigraphic and oxygen isotope evidence for My-scale glaciation driving eustasy in the Early–Middle Devonian greenhouse world. *Palaeogeography, Palaeoclimatology, Palaeoecology* **276**, 170–181 (2009).
52. Lenton, T. M. *et al.* Earliest land plants created modern levels of atmospheric oxygen. *Proc Natl Acad Sci U S A* **113**, 9704–9 (2016).
53. Lenton, T. M., Daines, S. J. & Mills, B. J. W. COPSE reloaded: An improved model of biogeochemical cycling over Phanerozoic time. *Earth-Science Reviews* **178**, 1–28 (2018).
54. Berner, R. A. GEOCARB II; a revised model of atmospheric CO 2 over Phanerozoic time. *American Journal of Science* **294**, 56–91 (1994).
55. Glasspool, I. J. & Scott, A. C. Phanerozoic concentrations of atmospheric oxygen reconstructed from sedimentary charcoal. *Nature Geosci* **3**, 627–630 (2010).
56. Sønderholm, F. & Bjerrum, C. J. Minimum levels of atmospheric oxygen from fossil tree roots imply new plant−oxygen feedback. *Geobiology* **19**, 250–260 (2021).
57. Porada, P. *et al.* High potential for weathering and climate effects of non-vascular vegetation in the Late Ordovician. *Nat Commun* **7**, 12113 (2016).
58. Halder, S., Arens, S. K. M., Jensen, K., Dahl, T. W. & Porada, P. A dynamic local-scale vegetation model for lycopsids (LYCOm v1.0). *Geosci. Model Dev.* **15**, 2325–2343 (2022).
59. Krause, A. J. *et al.* Stepwise oxygenation of the Paleozoic atmosphere. *Nat Commun* **9**, 4081 (2018).
60. Krause, S. *et al.* Microbial nucleation of Mg-rich dolomite in exopolymeric substances under anoxic modern seawater salinity: New insight into an old enigma. *Geology* **40**, 587–590 (2012).
61. Bergman, N. M. COPSE: A new model of biogeochemical cycling over Phanerozoic time. *American Journal of Science* **304**, 397–437 (2004).
62. McKenzie, N. R. *et al.* Continental arc volcanism as the principal driver of icehouse-greenhouse variability. *Science* **352**, 444–447 (2016).
