# Supplementary material for: Low atmospheric CO2 levels before the rise of forested ecosystems
Source: Nat Commun. 2022 Dec 20;13:7616. doi: 10.1038/s41467-022-35085-9 (PMC9768202; doi:10.1038/s41467-022-35085-9)
Supplement: Supplementary file 3 — Description of Additional Supplementary Files [file 41467_2022_35085_MOESM3_ESM.pdf]

Description of Additional Supplementary Files:

Supplementary Data 1: Carbon isotopic and stomatal data of Devonian plants; model input parameters and outputs from the gas-exchange model, including predicted palaeopCO_2, CO_2 assimilation rates (An) and water conductance (gc,tot) of fossil lycophytes with living descendants and other fossil plants (rhyniophytes, horneophytes, zosterophylls) with no living descendants. The data and results are summarized for individual fossil localities, where stomata density and pore size has been reported (references listed in Supplementary table 6) and for which carbon isotope data exists (Supplementary table 1 and Wan et al.[1]). The stomata and isotope data are derived from distinct fossil specimens, and therefore we adopt the 1 standard deviation variability among multiple specimens to account for the plant-to-plant variability. Uncertainties of the output parameters are asymmetric and calculated by Monte Carlo error propagation, as illustrated in supplementary fig. 6. The following two pages in the spreadsheet summarize results from fossil lycophytes and modern descendants (where A0 = 3.73 µmol/s/m^2). The fourth page summarizes estimated values for A0 for fossil plant of extinct lineages obtained by matching observations from lycophytes. The fifth page includes notes on where stomatal and isotopic data comes from the same palaeolocation (paired data) and notes on assumed best matching locations in the cases of unpaired data.

Supplementary Data 2: Palaeoclimatic conditions at fossil sites as predicted by our CLIMBER palaeoclimate model with an atmospheric CO_2 level of 500 ppm. Palaeoproxy temperature estimates derived from d18O composition of non-luminescent calcitic brachiopods and phosphatic conodonts in various sections and locations. Comparison between modelled sea surface temperatures and proxy data (assuming d18O of Devonian seawater was -1.1‰). A calculation of Devonian seawater d18O best compatible with paleotemperature proxy data and models with either 500 and 2000 ppm CO_2.
